# Dimorphic cocoons of the cecropia moth (*Hyalophora cecropia*): Morphological, behavioral, and biophysical differences

**DOI:** 10.1371/journal.pone.0174023

**Published:** 2017-03-22

**Authors:** Patrick A. Guerra, Steven M. Reppert

**Affiliations:** Department of Neurobiology, University of Massachusetts Medical School, Worcester, Massachusetts, United States of America; INRA-UPMC, FRANCE

## Abstract

The larvae of the giant silk moth (*Hyalophora cecropia*) spin strikingly dimorphic, multilayered cocoons that are either large and fluffy (baggy) or significantly smaller and tightly woven (compact). Although these cocoon-morphs share the same function (i.e., housing for pupal to adult development during overwintering), previous work has been unable to determine why cocoon dimorphism exists. We addressed this issue in cecropia moth cocoons collected along power line right-of-way habitats in Massachusetts. We first characterized the architectural differences between cocoon-morphs for all three cocoon sections (outer and inner envelopes, and the intermediate layer separating the two). We show that outer envelope structural and ultrastructural differences are what underlie dimorphism. Using a common spinning arena, we next show that the behavioral suites used to construct the outer envelopes of the two morphs are significantly different in behavioral time investment and patterning, as well as in the location of silk placement in the common spinning arena. Finally, we compared the cocoon-morphs in response to various environmental stressors to ask whether dimorphism is an adaptive response to such pressures. In contrast to compact cocoons, we find that baggy cocoons act as heat sinks and allow greater moisture permeability; differences in outer envelope architecture underlie these characteristics. These two biophysical properties could be advantageous for pupae in baggy cocoons, during unseasonably cold or dry conditions encountered during development prior to adult emergence. Our results suggest that cocoon dimorphism in the cecropia moth may provide a bet-hedging strategy for dealing with varying environmental conditions in Massachusetts and perhaps over its entire habitat range, during pupal to adult development.

## Introduction

The silk cocoons spun by individuals of many insect taxa [1–2], such as that by caterpillars of many moth species function as a protective exterior covering for individuals during their pupal stage, and from which individuals will later emerge (eclose) as adults. Despite sharing the same function, there is great variation in the morphology and level of architectural sophistication of cocoons across moth species. In general, caterpillars will spin a cocoon that is stereotyped and species-specific, such that it can be considered an extended phenotype of the individual [3]. These differences in cocoon morphology across species may reflect specific adaptations and different strategies to maximize survivorship or proper development during the pupal stage. This includes species-specific responses to different topographies or environmental conditions during cocoon construction.

**Fig 1:**
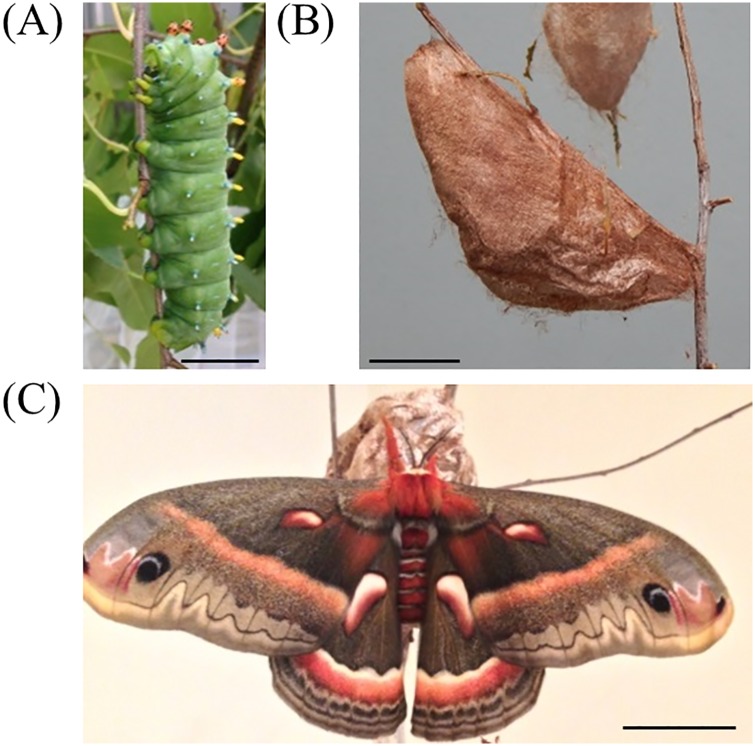
Life cycle of cecropia silk moth examined in this study. Late fifth instar caterpillars (A) spin a cocoon (B) during the summer, in which they pupate and overwinter. The adult moth emerges (C) from its cocoon in the following spring. For (A-C), black bars represent 2.54 cm.

**Fig 2:**
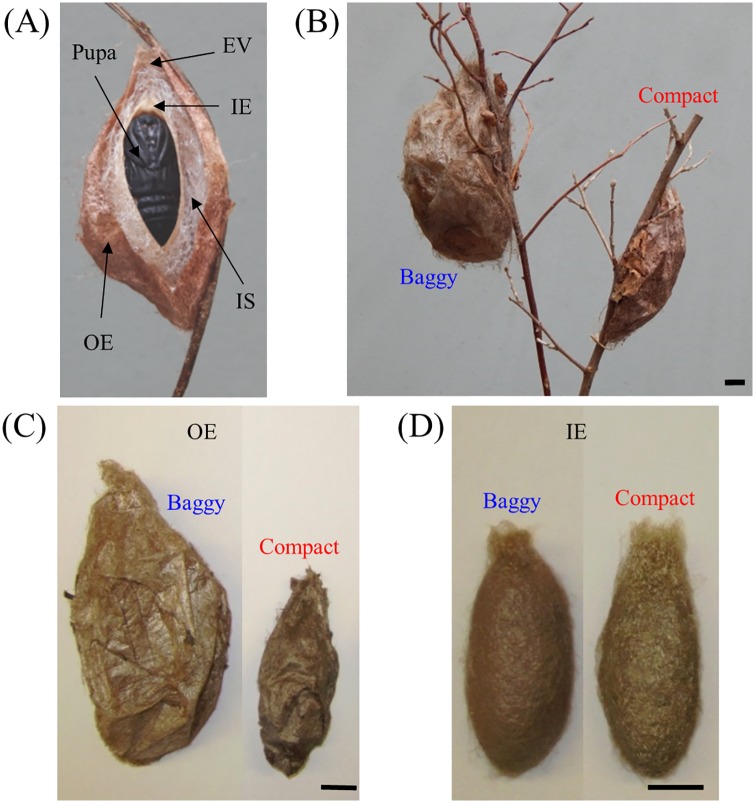
The dimorphic, multilayered architecture of cocoons constructed by larvae of the cecropia moth. (A) A cecropia moth cocoon possesses three distinct layers, consisting of an outer envelope (OE), an intermediate space filled with silk (IS), and an inner envelope (IE). Each cocoon is constructed with an escape valve (EV) from which adults can exit the cocoon. (B) A cocoon is constructed to be either a large, fluffy baggy morph (left) or a smaller, more tightly woven compact morph (right). (C) The OEs of baggy (left) and compact (right) cocoons are architecturally dimorphic. (D) The IEs of baggy (left) and compact (right) cocoons (pictured here are the IEs of cocoons contained within the OEs found in [C]), are equivalent architecturally. For (A-B) cocoons are shown attached to branches and still possessing leaves, whereas (C-D) cocoons are shown removed from branches and stripped of leaves. For (B-D), black scale bars denote 1 cm.

The cocoons spun by caterpillars of the cecropia silk moth, *Hyalophora cecropia* (Lepidoptera; Saturniidae) are examples of animal structures built with a high-level of architectural complexity. Spun by late fifth instar larvae during the summer, these cocoons serve as overwintering housing during the pupal stage and from which individuals will later eclose as adults in the following spring (Fig 1A–1C). After assembling an intricate silk scaffold which anchors the cocoon to the spinning site, a caterpillar constructs a multilayered cocoon that possesses outer and inner envelopes, two discrete layers separated by an intermediate space filled with silk [4–6]. As part of its architecture, a cocoon possesses valves (in both the outer and inner envelopes) from which the fully formed adult exits the cocoon. The escape valves are typically pointed upwards and oriented perpendicular relative to the ground or the horizontal plane (Fig 2A). Across the cecropia moth habitat range, a striking cocoon dimophism exists in which larvae can spin either a large, fluffy (baggy) cocoon or a significantly smaller, tighter (compact) cocoon (Fig 2B–2D).

For over a century [7], an intriguing evolutionary puzzle is why such striking cocoon dimorphism has evolved in the cecropia moth. In contrast to other examples of morphological dimorphism, such as flight wing polymorphism in insects [8] in which different structural morphs represent coexisting, alternative life history strategies, an adaptive function for cocoon dimorphism in the cecropia moth is still unknown [9]. We propose that cecropia moth cocoon dimorphism in the Massachusetts study population might represent a diversifying bet-hedging strategy [10–12]. As it is unknown which cocoon-type will be best suited for the conditions of the upcoming spring, the construction of different cocoon-types during the preceding summer spreads the risk and the negative impacts of unpredictable environmental conditions during pupal development [11–12].

For our study, we first examined the natural level of dimorphism of cecropia moth cocoons collected outdoors in the wild along power line right-of-way habitats in Massachusetts, and compared it to the level of dimorphism of cocoons constructed in both semi-natural outdoor and indoor conditions. We then conducted a detailed description of the structural and ultrastructural differences between the cocoon-types at all three layers of cocoon construction. We also examined cocoons using several biological markers that may underlie dimorphism. These included a response to different selection pressures, such as predation and parasitism, and correlation with the phenotypic traits of the animals that constructed the cocoons, such as their sex, size, and timing (time of year and time of day) of adult eclosion. We then used a common cocoon-spinning environment to show that both cocoon types can be spun under the same spinning conditions and site topography. These results allowed us to examine the behavioral differences used to produce the two cocoon-morphs. Finally, we showed that cocoon dimorphism leads to differences in the functional biophysical properties of the cocoon-types (e.g., temperature and moisture regulation). Our results suggest that cocoon dimorphism in Massachusetts cecropia moths may provide a bet-hedging strategy for dealing with varying environmental conditions during the period prior to adult eclosion. This strategy may exploit the differences in biophysical properties found between cocoon morphs.

## Results

### Cocoon dimorphism under different conditions

To measure the natural dimorphism of a population of cecropia moth cocoons from Eastern and Central Massachusetts, we analyzed 233 cocoons collected along power line right-of-way habitats from December to March of 2013–2016. Visual inspection (confirmed by morphometric measures; see below) showed that 136 were baggy and 97 were compact. Baggy cocoons thus represented 58% of the total in this collection. Although baggy cocoons are significantly larger than compact cocoons (see cocoon architectural analysis below), it is unlikely that their larger size biased our field sampling. In addition, we did not find clear differences in the location of cocoon morphs in the field; the height of spinning site off the ground was similar between the two (see below).

For the spring of 2014, our focal collection sample (see Methods), we found that there was a similar probability that a collected cocoon of the 89 total would be either baggy or compact (Chi-squared test: χ^2^ = 0.01, *P* > 0.9), with 49% of this collection being baggy. When this sample was compared to cocoons that were raised in both semi-natural outdoor (2015: Auburndale, Ma: *N* = 22 cocoons, 55% baggy; Lexington, MA: *N* = 140, 41%) and indoor conditions (2015: *N* = 26 cocoons, 55% baggy), we found no difference in the proportion of cocoons that were baggy across all groups (Fisher’s exact test, *P* > 0.5). A similar proportion of cocoons that were baggy across all groups suggests that the different rearing conditions that occurred outdoors (2014 and 2015) and indoors (2015) during our study had little influence on the occurrence of cocoon dimorphism. Moreover, preliminary analysis of cocoons from the offspring of a baggy x baggy mating (*N* = 22 cocoons, 55% baggy, 2015 semi-natural outdoor group from Auburndale, MA) did not increase the incidence of offspring spinning baggy cocoons, suggesting that a genetic polymorphism does not explain the dimorphism.

### Cocoon dimorphism design

**Fig 3:**
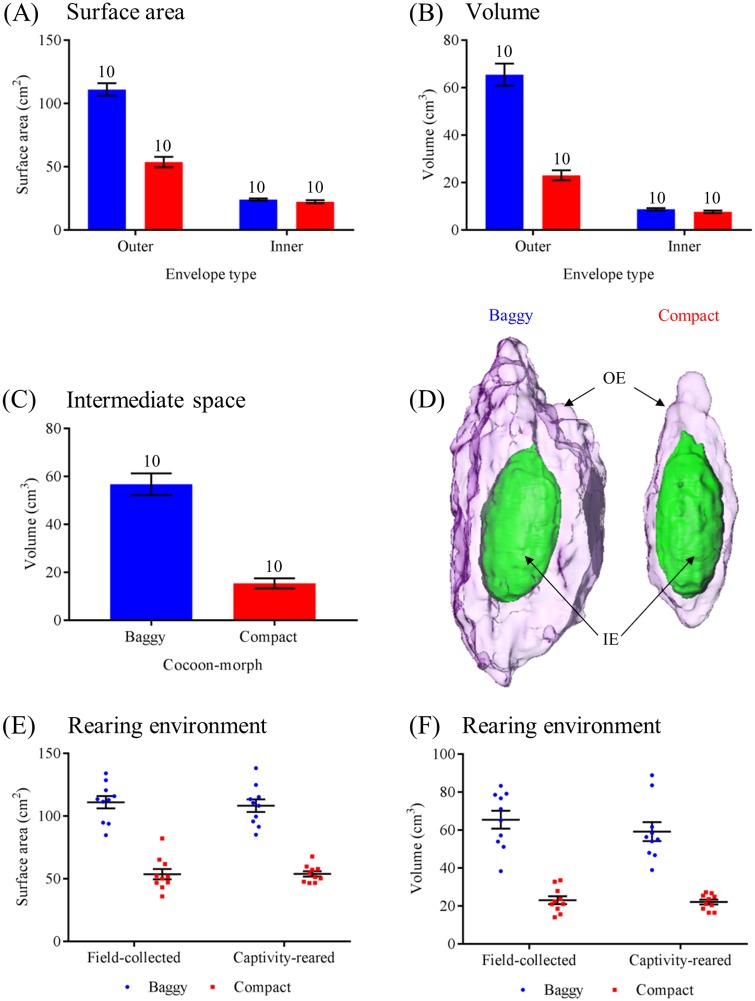
Structural properties of dimorphic cecropia moth cocoons. (A) Surface area comparisons of the outer envelope and inner envelope of baggy and compact cocoons. (B) Comparisons of the volume of the outer envelope and inner envelope of baggy and compact cocoons. (C) The volume of the intermediate space of baggy and compact cocoons. (D) The spatial relationship between the outer envelope (OE) and inner envelope (IE) of a baggy and a compact cocoon. (E) Comparing the surface area and volume (F) of baggy and compact cocoons that were either field-collected or spun in captivity. For all panels, blue denotes baggy cocoons and red denotes compact cocoons. For (A)–(C), sample sizes are 10 for each group. For (E) and (F), sample sizes are 10 for both cocoon-morphs, in both field-collected and captive-spun groups. Error bars denote standard errors of the mean.

Next, we analyzed the structural and ultrastructural difference between the two morphs of field-collected cocoons to confirm our gross visual observations, and to determine at what level of cocoon construction dimorphism occurs. Using 3D scanning techniques, we found that the outer envelope of baggy cocoons had greater surface area than the outer envelope of compact cocoons, whereas the inner envelopes of both cocoon-morphs were similar (Two-way ANOVA: *F*_(1, 36)_ = 70.56, *P* < 0.0001; Tukey post-hoc tests: baggy inner envelope vs. compact inner envelope, *P* > 0.9; *P* < 0.0001 for all other comparisons; Fig 3A). We also found that the outer envelope of baggy cocoons had greater volume than the outer envelope of compact cocoons, but there was no difference in volume between the inner envelopes of both cocoon-morphs (Two-way ANOVA: *F*_(1, 36)_ = 63.61, *P* < 0.0001; Tukey post-hoc tests: baggy inner envelope vs. compact inner envelope, *P* > 0.9; *P* < 0.01 for all other comparisons; Fig 3B). The intermediate space of baggy cocoons had significantly greater volume than the intermediate space of compact cocoons (Mann-Whitney test: *U* = 0.0, *P* < 0.0001; Fig 3C and 3D, S1 Video).

Both captive and field-collected cocoons had similar dimorphic features (Fig 3E), with baggy cocoons having greater surface area than compact cocoons in both environments (Two-way ANOVA: no interaction between environment and cocoon-morph, *F*_(1, 36)_ = 0.13, *P* > 0.7; no effect of environment, *F*_(1, 36)_ = 0.09, *P* > 0.7; difference between cocoon-morphs, *F*_(1, 36)_ = 173.4, *P* < 0.0001). Similarly, both groups had similar volume (Fig 3F), with baggy cocoons in both groups having greater volume than compact cocoons (Two-way ANOVA: no interaction between environment and cocoon-morph, *F*_(1, 36)_ = 0.54, *P* > 0.4; no effect of environment, *F*_(1, 36)_ = 0.99, *P* > 0.3; difference between cocoon-morphs, *F*_(1, 36)_ = 120.1, *P* < 0.0001). These quantitative structural measures thus verify our gross visual assessments of dimorphic cocoon type for both captive and field-collected cocoons.

**Fig 4:**
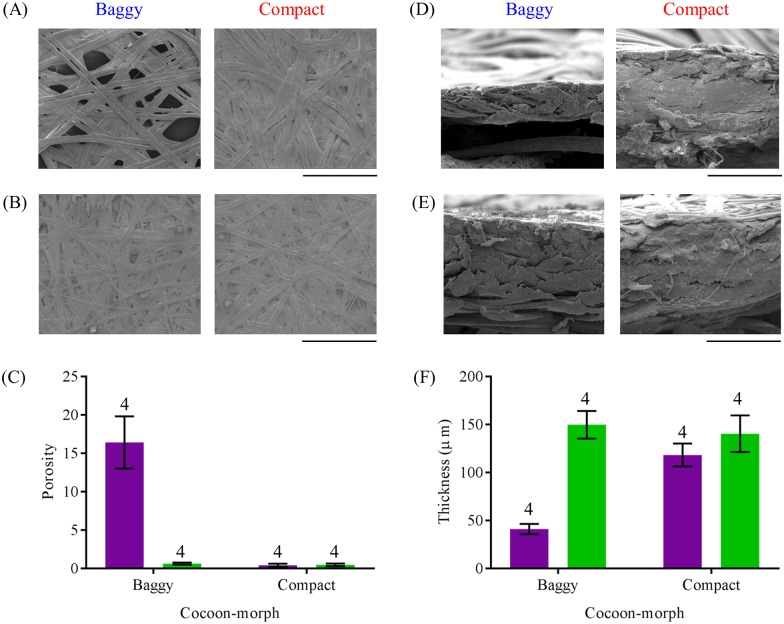
Ultrastructural properties of dimorphic cecropia moth cocoons. Flat SEM images (magnification: 175x) of the (A) outer envelope and of the (B) inner envelope of a baggy and compact cocoon. (C) Porosity estimate comparisons between the outer envelope and inner envelope of baggy and compact cocoons. Cross-sectional SEM images (magnification: 706x) of the (D) outer envelope and the (E) inner envelope of a baggy and compact cocoon. (F) Thickness comparisons between the outer envelope and inner envelope of baggy and compact cocoons. For (A) and (B), black bars represent 400 μm and for (D) and (E), black bars represent 100 μm. For both (C) and (F), *N* = 4 for all groups, and purple bars denote the outer envelope, and green bars denote the inner envelope, of baggy and compact cocoons. Error bars denote standard errors of the mean.

Using scanning electron microscopy (SEM), our ultrastructural analysis of field-collected cocoons showed that the outer envelope of baggy cocoons was more porous than the outer envelope of compact cocoons (Fig 4A), and that the inner envelopes of both cocoons-morphs had similar porosity (Fig 4B) (Two-way ANOVA: *F*_(1, 12)_ = 21.65, *P* < 0.001; Tukey post-hoc tests: baggy outer envelope vs. all other groups, *P* < 0.05; *P* > 0.05 for all other comparisons; Fig 4C). We also found that the outer envelope of baggy cocoons was thinner than the outer envelope of compact cocoons (Fig 4D), and there was no difference in thickness between the inner envelopes of both cocoon-morphs (Fig 4E) (Two-way ANOVA: *F*_(1, 12)_ = 10.12, *P* = 0.01; Tukey post-hoc tests: baggy outer envelope vs. all other groups, *P* < 0.05; *P* > 0.05 for all other comparisons; Fig 4F).

Baggy and compact cocoons also differed in how much silk is allocated to the outer envelope and to the silk found in the intermediate space. We found no difference in whole cocoon silk weight between baggy (0.78 ± 0.04 g, *N* = 10) and compact (0.68 ± 0.04 g, *N* = 10) cocoons (t-test: *t*_18_ = 1.82, *P* = 0.09), demonstrating that cocoons of both morphs are constructed with the same amount of silk. Compared to baggy cocoons (45.89 ± 2.32%, *N* = 10), compact cocoons (53.22 ± 2.11%, *N* = 10) allocated a greater percentage of a cocoon’s silk to the outer envelope (t-test: *t*_18_ = 2.34, *P* = 0.03). In contrast, baggy cocoons (13.36 ± 0.85%, *N* = 10) possessed a greater amount of silk in the intermediate space than compact cocoons (5.45 ± 0.84, *N* = 10) (t-test: *t*_18_ = 6.64, *P* < 0.0001). There was no difference in the amount of silk allocated to the inner envelope between the cocoon-morphs (t-test: *t*_18_ = 0.19, *P* > 0.8; baggy cocoons: 40.75 ± 2.03, *N* = 10; compact cocoons: 41.33 ± 2.25%, *N* = 10). Thus, our morphometric and silk allocation analyses show that the size difference between the two cecropia moth cocoon morphs lies mainly in the construction of the outer envelope.

### Cocoon dimorphism and viability

We also examined whether cocoon viability and/or height above ground contributed to cocoon dimorphism. Of the 89 cocoons collected in 2014, 23 cocoons (26%) were found to be viable, and there was no difference in the proportion of cocoons that were viable between baggy (13/44) and compact (10/45) cocoons (Fisher’s exact test, *P* > 0.4). For cocoons in which we obtained a height measurement (from off the ground to the spinning site), we found no difference in the mean height of cocoons between the cocoon-morphs (Unpaired t-test: *t*_(80, 45)_ = 0.09, *P* > 0.9; baggy: 50.8 ± 5.8 cm, *N* = 39; compact: 51.6 ± 6.7 cm, *N* = 44). 42% (37/89) of collected cocoons were physically damaged, with no difference in the proportion of cocoons with damage between the cocoon morphs (Fisher’s exact test: *P* = 1.00; baggy: 18/44; compact 19/45). 45% (40/89) of collected cocoons, regardless of cocoon-morph, were parasitized. There was no difference between the cocoon-morphs in the proportion that were parasitized (Fisher’s exact test: *P* = 0.06; baggy: 15/44; compact: 25/45).

### Cocoon dimorphism and adult eclosion

**Fig 5:**
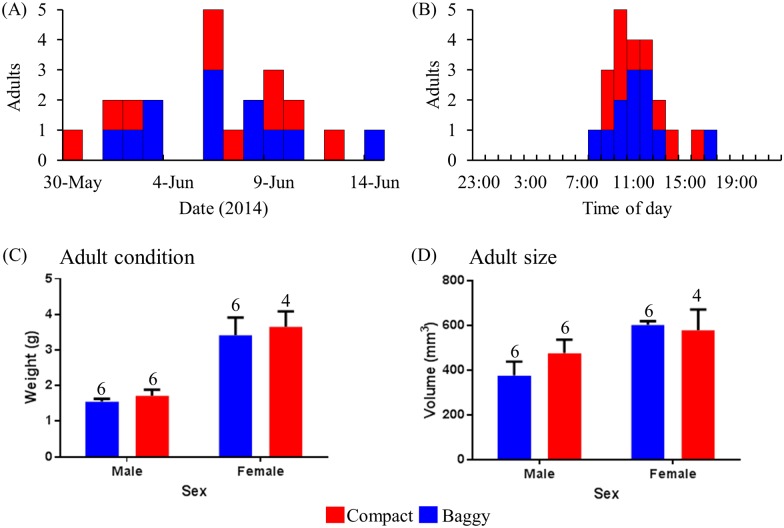
Emergence of adult cecropia moths from field-collected cocoons. (A) Emergence dates of adults from baggy and compact cocoons. (B) Daily adult emergence times of adults from baggy and compact cocoons. (C) Sex and condition (weight, g) of adults emerging from both baggy and compact cocoons. (D) Sex and size (thorax volume, mm^3^) of adults emerging from both baggy and compact cocoons. For all figures, blue bars are baggy cocoons and red bars are compact cocoons.

To examine whether the normal yearly and daily timing of adult eclosion by individuals contained within cocoons is altered by cocoon dimorphism, we compared the yearly temporal patterns and time of day of adult eclosion from both cocoon-types. The time of year when the cecropia moth ecloses from its cocoon is controlled largely by temperature, consisting of a period of cold dormancy followed by temperature dependent adult development [13]. As thermoregulation of cocoon morphs can vary (see below), we examined the time of year adult eclosion occurred to determine whether there was a difference between morphs. From viable cocoons examined in the spring of 2014, adults emerged between May 30^th^, 2014 and June 14^th^, 2014 in a unimodal pattern, and there was no difference in the mean date of emergence according to cocoon-morph type during this time period (Mann-Whitney test: *U* = 58.50, *P* > 0.9; baggy mean date: June 6^th^; compact mean date: June 6^th^; Fig 5A).

The daily timing of adult eclosion in the cecropia moth is governed by an individual’s circadian clock, which is entrained to prevailing lighting conditions [14–15]. Because less biologically relevant light for clock entrainment penetrates the compact cocoon compared to baggy (see below), the daily timing of adult eclosion could be altered between the two cocoon types. However, this was not the case: for both cocoon-morphs, adult emergence times were identical (Watson’s test: *U*^*2*^_(10, 12)_ = 0.07, *P* > 0.5) and significantly clustered at midday (baggy cocoons: mean time 12:31, Rayleigh’s test: *Z* = 8.75, *P* << 0.0001, *r* = 0.85, *N* = 12; compact cocoons: mean time 12:44, Rayleigh’s test: *Z* = 7.34, *P* << 0.0001, *r* = 0.86, *N* = 10; Fig 5B).

We also found no relationship between cocoon-morph and the sex of the adult that eclosed from a cocoon (Fisher’s exact test: *P* > 0.6; adult males: *N* baggy = 6, *N* compact = 6; adult females: *N* baggy = 6, *N* compact = 4). When adult condition was assayed using adult weight (g), we found no interaction between the sex of the adult and the cocoon that it emerged from (Two-way ANOVA: *F*_(1, 17)_ = 0.01, *P* > 0.9), and there was no difference in adult weight between those that emerged from the different cocoon-morphs (main effect: *F*_(1, 17)_ = 0.41, *P* > 0.5; Fig 5C). Similarly, when we examined adult size using thorax volume as a proxy for size, we found no interaction between the sex of the adult and its cocoon type (Two-way ANOVA: *F*_(1, 17)_ = 0.99, *P* > 0.3), and no influence of adult size on cocoon type (main effect: *F*_(1, 17)_ = 0.37, *P* > 0.5; Fig 5D).

### Dimorphic cocoon construction behavior

**Fig 6:**
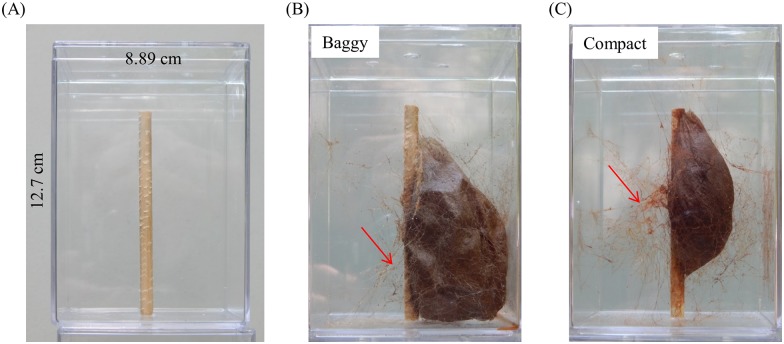
Common spinning arena used to assay dimorphic cocoon construction. (A) Common spinning arena used to assay cocoon construction. Paper grids on walls of arena omitted for clarity (see Methods). Both baggy (B) and compact (C) cocoons were constructed in our common arena apparatus. In both (B) and (C), red arrows indicate silk scaffold assembly for each cocoon.

Given the marked differences in the structural and ultrastructural nature of the outer envelope between cocoon morphs, we next examined whether there are behavioral differences between the construction of baggy and compact outer envelopes that explain the dimorphism. To perform such behavioral analysis, we developed a common spinning arena (Fig 6A and see S2 Video) in which both morphs were consistently spun in captivity (baggy cocoon: Fig 6B; compact cocoon Fig 6C).

In our spinning arena trials, baggy cocoons (679.6 ± 23.09 cm^3^, *N* = 5) had significantly greater volume (square grid measurements) than compact cocoons (283.2 ± 20.97 cm^3^, *N* = 13) (t-test: *t*_16_ = 10.70, *P* < 0.0001), demonstrating that cocoon-dimorphism is maintained under controlled conditions. These data confirmed that lab-spun cocoons are indistinguishable from those found in nature.

**Fig 7:**
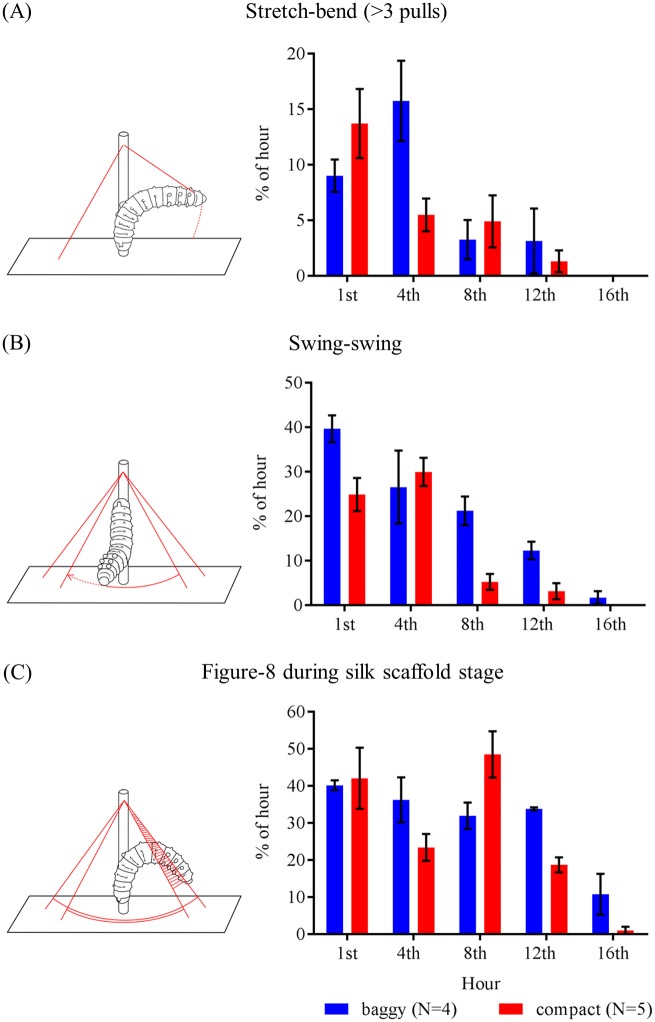
Different construction behavioral patterns produce dimorphic cocoons in *H*. *cecropia*. Bar graphs show time budget comparisons between baggy (blue) and compact (red) cocoon spinners for (A) stretch-bend behavior (> 3 pulls), (B) swing-swing behavior, and (C) Figure-8 silk laying, during the first 18 hours of cocoon spinning (see S2 Table for statistical analyses). Error bars denote standard errors of the mean. For (A-C), the cartoon to the left of each bar graph depicts the silk-spinning behavior of the caterpillar for that particular behavior. Solid red lines indicate silk and dashed red lines denote the movement of the caterpillar; structure perpendicular to arena floor (rectangle) is the dowel found in the spinning arena. Cartoons were re-drawn from [16] by Daniel J. Newman.

**Fig 8:**
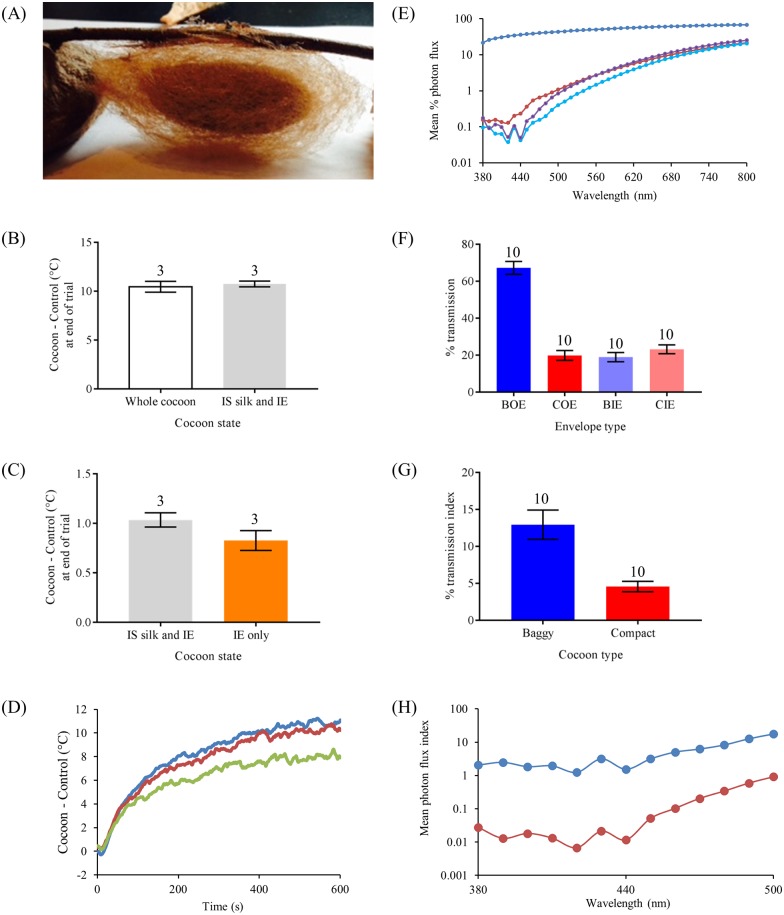
The spectral properties of cecropia cocoons. (A) An example of a baggy cocoon with only its inner envelope and its silk found in the intermediate space (attached to the inner envelope). (B) Baggy cocoons have no difference in infrared (IR; wavelengths > 700 nm) heating with or without the outer envelope. (C) The enhanced IR heating effect of baggy cocoons requires the presence of silk in the intermediate space. For both (B) and (C), intermediate space is designated by IS, and inner envelope is denoted by IE. (D) Heating curves of a baggy cocoon demonstrating the role of the silk in the intermediate space for IR heating (blue: whole cocoon; red: silk in the intermediate space and inner envelope only; green: inner envelope only). (E) Mean irradiance curves (*N* = 10 for each type) measuring light transmittance through the different envelopes of baggy (outer envelope: blue; inner envelope: light blue) and compact (outer envelope: red; inner envelope: purple) cocoons. Error bars omitted for clarity. (F) Comparison of percent transmittance of IR light wavelengths through the different envelope types of baggy (outer envelope: BOE; inner envelope: BIE) and compact (outer envelope: COE; inner envelope: CIE) cocoons. (G) Comparison of transmittance of IR light into the interior of the cocoon between baggy and compact cocoons. (H) Transmittance of blue light wavelengths into the interior of the cocoon for both baggy and compact cocoons. Sample sizes for each group in (F)–(H) are *N* = 10. Error bars denote standard errors of the mean.

Using a 3D recording system (see Methods), our behavioral analyses found overall that baggy and compact cocoons share a similar construction process, in which both cocoon-morphs are spun through various stages using distinct spinning behaviors previously described [5]. In fact, caterpillars employ a similar repertoire of the three main behaviors, stretch-bend, swing-swing, and figure-8, for constructing either cocoon-morph (all behavioral types are defined in S1 Table; see Fig 7 for diagrams of main behaviors).

Despite the similar stages of progression during cocoon construction, we found significant differences in the time invested in the different cocoon construction behaviors (Fig 7, S1 Appendix, and S2 Table). Baggy spinners spend more time performing the three main cocoon construction behaviors than compact spinners (see Fig 7 and S2 Table), and there are additional differences between baggy and compact spinners for the other cocoon construction behaviors (see S2 Table for analyses of the other cocoon construction behaviors).

We also observed differences in the sequences and patterns of spinning behaviors (measured using ethograms; S2 Appendix and S1 Fig) performed during construction, and differences in the locations where spinning behaviors were performed in the arena (measured using XYZ scatterplots; S3 Appendix and S2 Fig), between the two cocoon-morphs during both silk scaffold and outer envelope construction stages. In contrast, we found no difference between baggy and compact spinners in the number of 180° (Two-way repeated measures ANOVA: *F*_(15, 105)_ = 0.6369, no interaction of main effect, *P* > 0.3) and 360° (t-test: *t*_7_ = 0.64, *P* > 0.5) body turns in trials. These results show that caterpillars are oriented in a similar manner when depositing silk at the common spinning site, even if the outcome cocoon (silk scaffold and outer envelope) is different between individuals.

### Functional properties of dimorphic cocoons

#### Thermoregulation

A difference in thermoregulation between the cocoon morphs may provide a selective advantage of one morph over the other. We found that baggy cocoons acted as greater heat sinks than compact cocoons in thermoregulation trials measuring either heat-gain or heat-loss, using two types of heat sources (either convection or infrared radiation, IR [wavelengths > 700 nm]).

In heat-gain convection trials, intact baggy cocoons had higher internal temperatures than intact compact cocoons. In contrast, without the outer envelope and the silk from the intermediate space, there was no difference in internal temperature between the inner envelopes of the two cocoon-morphs (see S4 Appendix and S3 Table). In heat-loss convection trials, however, intact baggy and compact cocoons exhibited similar thermoregulatory behavior (S4 Appendix and S3 Table).

In trials in which we used IR as a heat source, intact baggy cocoons had higher internal temperatures (heat-gain trials) and retained more heat (heat-loss trials) during the trial period than intact compact cocoons (Fig 8, S4 Appendix, and S3 Table). We found that the enhanced IR heating effect observed in baggy cocoons was due to two properties not exhibited by compact cocoons. First, baggy cocoons have a significantly greater amount of silk in the intermediate space (which was heated by IR light, Fig 8A). More importantly, this intermediate space silk of baggy morphs was necessary for the enhanced IR heating effect (Fig 8B–8D). Second, a greater amount of IR, which heats the silk found in the intermediate space, can pass through the more porous character of the baggy cocoon outer envelope (see Fig 8E–8G, S4 Appendix, and S3 Table for further details). The characteristics of baggy cocoons that allow them to absorb more heat (via convection and IR heating) than compact cocoons, may benefit baggy-contained individuals when developing under colder than normal spring conditions prior to adult eclosion.

#### Penetration of short-wavelength light in cocoon morphs

As previously noted, we found no evidence of cocoon dimorphism affecting the time of day of adult eclosion in the individuals contained within the different cocoon-morphs. Accordingly, we found that blue light (380–500 nm), light wavelengths necessary for the proper entrainment and synchronization of the circadian clocks in the pupal brain that regulate adult eclosion times [14, 15, 17, 18], can be similarly transmitted into the interior of the cocoon of both cocoon-morphs (Fig 8H). Baggy cocoons, however, have higher photon flux indices than compact cocoons between 380–500 nm, showing that light of greater intensity can reach the interior of baggy cocoons as compared with the interior of compact cocoons (Fig 8H). However, this difference in light intensity has no apparent effect on adult eclosion times for the cocoon-morphs (Fig 5A and 5B; as above).

#### Moisture permeability

**Fig 9:**
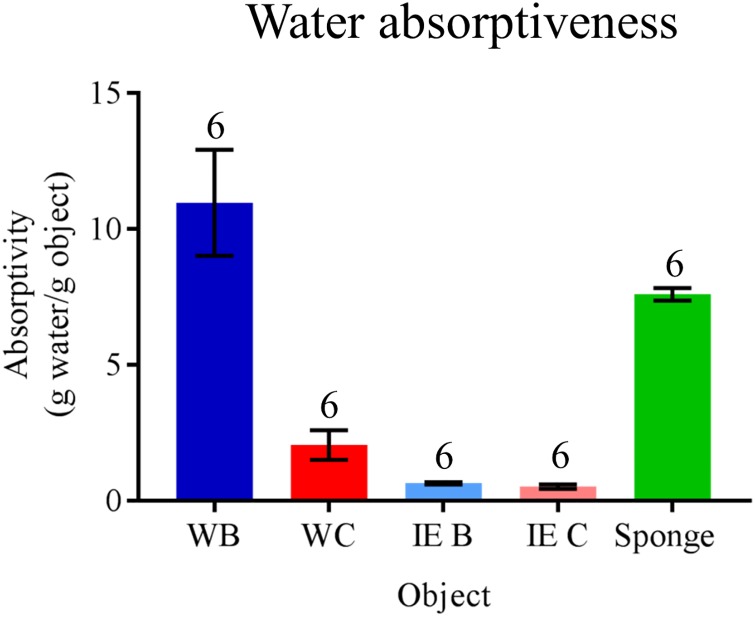
Water absorptiveness of baggy and compact *H*. *cecropia* cocoons. Whole baggy: WB, blue bar; Whole compact: WC, red bar; baggy with inner envelope only: IE B, light blue bar; compact with inner envelope only: IE C, pink bar; sponge (control): green bar. *N* = 6 for each group. Error bars denote standard errors of the mean.

Baggy and compact cocoons possessed markedly different levels of moisture permeability, as demonstrated in our water absorptiveness trials comparing cocoon-morph water absorption indices (One-way ANOVA: *F*_(4, 25)_ = 26.49, *P* < 0.001; Fig 9). This difference in moisture permeability only occurred when the cocoon-morphs were intact. Here, intact baggy cocoons absorbed water as readily as our control commercial sponges, whereas intact compact cocoons absorbed significantly less water than both intact baggy cocoons and controls (Tukey post-hoc tests, *P* > 0.05 for all comparisons; Fig 9). When cocoons were re-tested after the removal of the outer envelope and intermediate space silk, we found no difference in water permeability between the baggy and compact inner envelopes (Fig 9). In addition, we found no evidence of capillary action in cocoons in any of our trials (Fisher’s exact test, *P* < 0.0001). In contrast to control sponges that were wet throughout despite only being half-submerged in water during trials, cocoons in all trials were only wet in the section that was submerged in water. These results show that the outer envelope of baggy cocoons is what facilitates increased water absorption. The ability of baggy cocoons to absorb more moisture than compact cocoons may potentially be beneficial to pupae, such as during drought conditions during development (e.g., avoid desiccation).

## Discussion

We found that architectural dimorphism in cocoons made by caterpillars of the cecropia moth is manifested at the level of the outer envelope, in which baggy and compact cocoons differ from one another in both structural [4] and ultrastructural characteristics. We also show that baggy and compact cocoon morphologies are accompanied by their own separate behavioral program used for cocoon construction. These two programs each involve the assembly of a silk scaffold that acts as the necessary template for an outer envelope with either baggy or compact architectural properties. As cocoons of both cocoon-morphs contain the same amount of silk, what underlies outer envelope dimorphism is the different way that individuals position silk in the 3D space of the spinning site during construction. Our results suggest that the construction of different cocoon architectures does not require different or specific conditions to occur, at least after placement in the spinning arena. Finally, we determined that the morphological differences between baggy and compact outer envelopes produce cocoons which differ in thermoregulatory performance, spectral characteristics, and moisture permeability.

Morphological dimorphism is widespread in the animal kingdom. In many insect species, conspecifics can display sexual, seasonal, phase, or caste-specific morphological dimorphism [19–20]. An example of morphological dimorphism in which different morphs co-exist, and which are expressed in both males and females simultaneously, is wing polymorphism in insects [8, 21–22]. Cocoon dimorphism in cecropia moths is an example of morphological dimorphism that most closely resembles insect wing dimorphism, because both cocoon-morphs are constructed and co-exist under the same environmental and habitat conditions, and are produced by both males and females. However, in contrast, the results of our studies demonstrate that the factors (intrinsic and extrinsic to the individual) which may influence the spinning of one cocoon-type over another are more complex than that for wing dimorphism.

The cecropia moth is known to be a fugitive species constantly searching for successional habitats [23]. In our collection of cocoons from Massachusetts, all were found on successional habitats along power line right-of-ways. Power line companies maintain these pathways by cutting back overgrowth and encouraging successional (edge) habitats of shrubs and small trees with the pathways bordered by mature forest. We have found that in the successional habitats under power lines there is an equal probability of a cecropia moth larva spinning either a compact or baggy cocoon. In contrast to the Northeastern US, cecropia moth cocoons are largely found in successional urban habitats in new residential areas in the Midwestern US [23]. Moreover, prior studies have shown that the vast majority (>99%) of cecropia moth cocoons spun in Midwestern successional urban habitats are compact (e.g., [9]). This occurs even though most compact cocoons on twigs and branches of urban saplings (>85%) were attacked by birds [24].

The lack of a major influence of habitat structure on cocoon dimorphism in cecropia moths from Massachusetts may represent a diversified bet-hedging strategy in response to environmental uncertainty [25]. Here, bet-hedging involves the spinning of either the baggy or compact cocoon-morph, as it is unclear which of these two cocoon phenotypes will be suited for future environmental conditions, and which cocoon phenotype will provide individuals contained within, the greater fitness in the future. Given the large, extensive range of cecropia moths in North America (westward from the Atlantic Ocean to the Rocky Mountains, southwards from southern Canada to the Gulf of Mexico [26]), individuals from different populations within this range can face varied and unpredictable environmental conditions while in the cocoon (e.g., fluctuating temperature and moisture conditions that may affect adult development and emergence). Additionally, since the 1950’s, loss of habitat development, pesticides, the use of mercury-lamp lights, and parasitism have collectively led to a substantial decrease in the number of cecropia moths and other saturniid moths throughout their range [27] and these issues complicate interpretation of locale differences in cocoon type.

Nonetheless, it is possible that differences in the biophysical properties that we observed between the cocoon-morphs in our studies, may play a role in this potential bet-hedging strategy that we propose for the Massachusetts population in which the different cocoon-morphs hedge against stochastic environmental conditions. For example, we observed that as compared with compact cocoons, baggy cocoons can absorb more heat via convection, function as significantly greater IR heat sinks, and are more permeable to moisture. The advantage of baggy cocoons in these three biophysical traits is facilitated by the specific architectural features of the outer envelope of baggy cocoons relative to that of compact cocoons. As a heat sink, baggy cocoons may be able to counteract the effects of colder than normal spring conditions (which can retard development). If the thermoregulatory and moisture permeability advantages baggy cocoons possess in relation to compact cocoons do not convey a selective advantage for the individuals contained within cocoons, cocoon dimorphism may simply be a neutral trait that is tolerated across different environments [28]. Experiments are needed to determine if these differences in biophysical properties between the cocoon-morphs are adaptive.

Cecropia moth cocoon dimorphism would be consistent with a diversified bet-hedging strategy if a single genotype produces either the baggy or compact cocoon-phenotype when individuals received the same cues and/or are exposed to the same environmental conditions [25]. Our results with individuals from Massachusetts appear to fulfill some of these conditions. For example, the ability of siblings from baggy x baggy parents to construct either a baggy or compact cocoon in similar proportion (2015 semi-natural outdoor group) suggests that cocoon dimorphism is not due to a genetic polymorphism between the morphs. Individuals clearly spun either cocoon-type despite being tested under the same environmental conditions and with identical cocoon site topography in our spinning trials. Thus, there do not appear to be allelic, epigenetic, and/or environmental differences that play a major role in the expression of the cocoon phenotype. Moreover, we found with a similar probability of baggy and compact cocoons in the field (2014 spring field-collected cocoons), in the same habitat conditions and with cocoons presumably spun under the same environmental conditions. Future studies, however, from an array of parental crosses and with individuals from different geographical locations are needed to further assess the potential influence of genetic background on cecropia moth cocoon dimorphism.

In light of contemporary environmental stressors such as climate change, which can increase the probability of extreme weather events, increase the occurrence and duration of atypical colder temperatures during spring, and cause prolonged drought conditions, cocoon dimorphism can provide certain cecropia moth individuals with the ability to cope with increasing environmental changes. Experiments are now needed to determine how cocoon dimorphism, a trait that is consistent with a locale-dependent, bet-hedging strategy, may function in the cecropia moth. In particular, systematic experiments specifically comparing the fitness and survivorship of individuals of the different cocoon-types under different climatic conditions and in different locales in the field are now necessary.

## Materials and methods

### Cocoons

To obtain an overall measure of cocoon dimorphism in the field, cocoons were collected in the field from December to March of 2013–2016, at various locations characterized by new growth, mid- to low-lying shrubs found in disturbed areas along trails and power lines, and in successional habitats, in Central and Eastern Massachusetts. For our analyses which compared the different cocoon-types (see below), we used cocoons collected in 2014 as our focal sample. To generate caterpillars for assaying cocoon spinning behavior (see below), adults that emerged from cocoons collected in 2015 were mated. Cecropia silkmoths are neither endangered nor a protected species; no specific permits were required from the authorities in order to collect and use specimens in our study. Prior to removing a cocoon from its spinning site, its distance from the ground was measured.

We also examined cocoons that were spun in either semi-natural outdoor or indoor conditions during 2015. Semi-natural outdoor conditions consisted of caterpillars spinning cocoons on cut cherry tree branches (*Prunus*) that were sleeved in mesh bag cages, with cages kept outdoors. Caterpillars were kept in these outdoor cages from hatching to the completion of cocoon spinning. Cocoons were spun under these conditions at Auburndale, Newton, MA (latitude 42° 20’ N, longitude 71° 14’ W), and at Lexingtion, MA (latitude 42° 27’ N, longitude 71° 13’ W). For cocoons spun in indoor conditions, caterpillars that had purged their gut and that were ready to spin were removed from the outdoor mesh cages, and introduced into clear, plastic boxes (8.89 x 8.89 x 12.7 cm; US Acrylic) and kept indoors for the entire cocoon spinning process in Auburndale, Newton, MA. We compared the proportion of cocoons that were baggy from these semi-natural outdoor and indoor groups, with our cocoons collected in the field in 2014 (our focal group).

### Architectural properties of cocoons

We used cocoons collected in the field during spring 2014 for examining the architectural properties of baggy and compact *H*. *cecropia* cocoons. Prior to examining their architectural properties, we removed any leaves and branches that remained attached to a cocoon after removal from its outdoor spinning site, and once the adult had emerged from the cocoon. We used three-dimensional (3D) scanning to assess the structural properties of the different cocoon sections of both baggy and compact *H*. *cecropia* cocoons. We used a MakerBot Digitizer 3D scanner (MakerBot Industries) to obtain a 3D scan for each cocoon. For each cocoon, we produced scans for both its outer envelope and inner envelope: after scanning the whole cocoon to obtain an outer envelope scan, an inner envelope scan was obtained by removing the outer envelope and any silk contained within the intermediate space, after the outer envelope scan was made. Each 3D scan was then imported into the program netfabb Basic (Version 5.2.0, Autodesk, Inc.) to obtain surface area and volume measurements for both the outer envelope and inner envelope of each cocoon. The volume of the intermediate space of each cocoon was calculated by subtracting the inner envelope volume from that of the outer envelope volume. As the outer envelope obscures the inner envelope from view, to compare the undisturbed physical relationship between the outer envelope and inner envelope of baggy and compact cecropia cocoons, we used x-ray microtomography (microCT) to produce 3D images of unmanipulated cocoons. We obtained microCT tomographs for each cocoon using a SCANCO vivaCT 75 microCT scanner (Musculoskeletal Center Imaging Core, University of Massachusetts Medical School). The tomographs for each cocoon were imported into the program Amira (Version 5.2.1., Visage Imaging, Inc.), and were used to reconstruct a 3D image for the entire undisturbed cocoon. Once we completed the 3D scans, we weighed the silk that comprised the outer envelope and inner envelope, and the silk that was contained within the intermediate space, for each cocoon.

To assess the ultrastructural properties of baggy and compact cecropia cocoons, we obtained scanning electron microscope (SEM) images for the outer envelope and inner envelope of cocoons. For each cocoon used for SEM imaging, two 16 mm^2^ sections from the intersection of the vertical and horizontal midline points for both the outer envelope and inner envelope were removed using fine scissors. One of the 16 mm^2^ sections was used for taking flat SEM images, while the other section was used for cross-sectional SEM images. Samples were mounted on carbon tape that was affixed onto aluminum stubs. The samples were then carbon-coated using a Denton Vacuum 502-B carbon evaporator, followed by sputter coating with gold-palladium using a Cressington 208hr sputter coater. These coated samples were transferred to a SEM (FEI Quanta 200 FEG MKII, Core Electron Microscope Facility, University of Massachusetts Medical School) for observation. We used the image processing software package Fiji [29] to obtain porosity (flat samples) and thickness (cross-sectional samples) measurements from the SEM images.

### Demographics

Field-collected cocoons from 2014 were used in our demographics analysis. After collection, each cocoon was classified as having either baggy or compact morphology, examined for physical damage (e.g., holes from avian predators, tearing by terrestrial predators) and for evidence of parasitism (e.g., parasitism by both larval or pupal parasites that would preclude normal adult emergence), and pupal viability was assessed via light manual shaking of the cocoon (e.g., viable pupae within a cocoon make a light thudding noise when shaken; [30]). After examination, all collected cocoons were then maintained outdoors in screen cages, under natural conditions in Auburndale, Newton, MA. The date, time of day, and sex of adults emerging from these field-collected cocoons were recorded. After exiting the cocoon, each adult was placed at 4°C until it was immobile to facilitate weighing, with adult weight used as an indicator for condition. As a correlate for the size of each adult, the length, width, and height of the thorax for each animal were measured using fine calipers, in order to calculate thorax volume.

### Functional properties of cocoons

We compared the thermoregulatory, spectral, and moisture permeability properties of baggy and compact cocoons using field-collected cocoons obtained in spring 2014. For all cocoons tested, the individual contained within it had already emerged out of the cocoon in its adult form prior to testing. All trials for each experiment described below were conducted in an isolated room under controlled conditions (21°C, 22% relative humidity) at the University of Massachusetts Medical School. Convection thermoregulation trials were done in complete darkness. For IR thermoregulation and spectral property trials, the only source of light during a trial was the IR light and the test light source, respectively.

#### Thermoregulation

To assess the thermoregulatory properties of a cocoon, we inserted a thermistor (TA-29, Warner Instruments) inside a cocoon through the cocoon’s valve, to record its internal temperature across different temperature regimes, at the position in which a quiescent pupa would be found, i.e., inside the inner envelope and enveloped by the silk of the intermediate space and the outer envelope. As a control, and for direct comparison with the thermistor contained within a cocoon, we measured the temperature immediately outside of the cocoon using a second thermistor (distance between thermistors: 14 cm). For hot and cold temperature convection experiments (see below), each thermistor was connected to a separate channel of a TC-344B Dual Channel Heater Controller (Warner Instruments), to record the temperature readings of the thermistors during trials. The temperature data from each thermistor channel were digitized by connecting the heater controller unit to a data acquisition unit (Micro 1401, Cambridge Electronic Design Limited) that was then connected to a computer. Temperature data from the cocoon and control thermistors were acquired for plotting and analysis using Spike2 software (Version 6.10, Cambridge Electronics Design Limited). For infrared (IR) heating experiments (see below), each thermistor was connected to its own temperature monitoring unit (TM-3 Three-Scale Temperature monitor, Warner Instruments). Each monitoring unit was then connected to the data acquisition unit on separate channels, and data were digitized and acquired for analysis in the same manner.

#### Convection experiments: Temperature gain or loss in dimorphic cocoons

To test and compare the thermoregulatory properties of baggy and compact cocoons, we tested the thermoregulatory behavior of cocoons in response to acute, rapid thermal changes as done previously with cocoons of other moth species [31]. We generated two types of thermal conditions for our trials. To produce a hot temperature condition, we used a Percival incubator (Model I36-LL, Percival Scientific, Inc.) set to a constant 39°C. The ambient temperature of the trial room (21°C) was used as an ambient temperature condition.

For each trial, were first recorded the temperature of the cocoon and control thermistors in the ambient temperature condition for five minutes to establish a baseline. The cocoon and control thermistors were then simultaneously transferred into the hot temperature treatment condition, and we recorded temperatures for a testing period that lasted one hour. After this hour, we then returned the cocoon and control thermistors to ambient temperature conditions and recorded temperatures for one hour. We tested whole cocoons and cocoons with only the inner envelope remaining in both ambient temperature and hot temperature conditions. Using this protocol, we were able to produce conditions to test heat gain (ambient to hot) and heat loss (hot to ambient) for the two cocoon-morphs.

For each trial, we generated either a heating (heat gain) or cooling (heat loss) temperature curve as a measure of the thermoregulatory behavior of the cocoon in the trial condition, in which we plotted the difference in temperature between the cocoon and control thermistors for the duration of the trial. From each curve, we acquired the lag time for the start of heating or cooling, we measured the area under the curve (AUC; a measure of the temperature inside the cocoon relative to the exterior), the maximum temperature reached in heating trials, and the final temperature reached at the end of both heating and cooling trials. Using modeling techniques [32], we estimated k constants for each curve (heating and cooling rates) and produced a predicted temperature for each trial, if trials were to continue indefinitely.

#### Infrared heating: Temperature gain or loss in dimorphic cocoons

To examine the thermoregulatory properties of baggy and compact cocoons in response to IR heat, we positioned an IR heat lamp (250 W Prism red IR heat lamp, Halco Lighting Technologies) 40 cm directly over a cocoon containing a thermistor and a control thermistor, and recorded the temperature from the two thermistors [33–34]. Each trial consisted of recording the ambient temperature in the room for five minutes to establish a baseline reading for both the cocoon and control thermistors. After this baseline period, we turned on the IR lamp and recorded temperatures for 10 minutes. At the end of these 10 minutes, we turned off the IR lamp and continued to record temperature for another 10-minute period. Our pilot trials determined that the 10-minute period of IR exposure was sufficient to observe a temperature response by the thermistor in the cocoon, i.e., heating. We first conducted trials with whole baggy and whole compact cocoons. As we found that whole baggy cocoons had significantly higher internal temperatures than whole compact cocoons under IR (see Results), we tested baggy cocoons collected in spring 2015 from the same areas cocoons were collected in during spring 2014, in two other IR trial conditions using the same treatment protocol. The first condition consisted of these baggy cocoons being tested with their outer envelope removed, but with the silk of the intermediate space remaining attached to the inner envelope; the second condition had cocoons tested in which the silk of the intermediate space was removed, and only the inner envelope remained. Temperature data from all three of these IR experiments were analyzed as in our convection experiments.

#### Spectral properties of dimorphic cocoons

We compared the spectral properties of baggy and compact cocoons by measuring the intensity and quality of light that was transmitted through each cocoon layer separately. To make irradiance measurements, we positioned a light source (Utilitech 250W no. 0320778; spectrum: peak at 600 nm, range: 350–800 nm; intensity: 7.45 × 10^15^ photons s^−1^ cm^−2^) above a spectrometer (Ocean Optics USB 2000 fiber optic spectrometer) held vertically in place. We then positioned the middle section of either the outer envelope or inner envelope, i.e., a similar location in the envelope in which a section was removed for SEM imaging (see above) immediately on top, covering, and in contact with the spectrometer probe, and measured the light transmitted through the cocoon layer. Each cocoon irradiance measurement was accompanied by a control irradiance measurement in which the spectrometer probe was unobstructed during light measurement. For each cocoon’s outer envelope and inner envelope irradiance measurements, we calculated the percentage photon flux (envelope photon flux divided by the control photon flux) within 10 nm wavelength bins, along the spectrum of the light source used (380–800 nm), to determine the amount and quality of light transmitted through each the outer envelope and inner envelope. For each cocoon, we also calculated a photon flux index (outer envelope photon flux multiplied by inner envelope photon flux) within each 10 nm wavelength bin, as a proxy for the amount and quality of light that potentially can be transmitted from outside of the cocoon to the pupa contained within it.

#### Moisture permeability of dimorphic cocoons

We compared the moisture permeability of baggy and compact cocoons by comparing their performance in water absorption trials (modified from [35]). Prior to trials, whole cocoons were weighed to obtain a pre-trial weight. Trials consisted of half-submerging the whole cocoon in a beaker of water (400 mL of water) for 10 minutes. After 10 minutes, the cocoon was removed from the beaker, and the cocoon and water contained within it were weighed. As a proxy for moisture permeability, we calculated a water absorption index for each whole cocoon, by subtracting the initial dry cocoon weight from the weight of the tested cocoon (cocoon and absorbed water), and then dividing this value by the original cocoon dry weight. We also examined whole cocoons for evidence of capillary action during the trial. After trials, whole cocoons were allowed to dry completely for six days, prior to removing the outer envelope and silk within the intermediate space for testing the inner envelope of cocoons in isolation. The inner envelope of each cocoon was then tested in the same way in order to calculate a water absorption index for the inner envelope. For all whole cocoon and inner envelope trials, a positive control was conducted simultaneously for each baggy and compact cocoon tested. Positive controls consisted of half-submerging a sponge (6.5 x 6.0 x 2.0 cm; Scotch Brite) and calculating a water absorption index in the same manner. We also observed the sponges for capillary action during trials. We compared the water absorption indices and the occurrence of capillary action across groups (whole baggy, whole compact, baggy inner envelope, and compact inner envelope) using a one-way ANOVA and Fisher’s exact test, respectively.

#### Cocoon construction behavior

In spring 2015, adults that emerged from field-collected cocoons were mated with each other to generate caterpillars for trials to assay cocoon spinning behavior under controlled laboratory conditions. Two distinct mating pairs (male and female each from a field-collected baggy cocoon) were produced from these field-collected cocoons. An additional mating pair was produced by using a lab-reared adult female contained within an outdoor enclosure to attract a wild-drawn sexually-receptive male. All rearing of progeny from these mating pairs (from the egg to the 5^th^ and final instar) was conducted outdoors in mesh cages in Auburndale, Newton, MA, with caterpillars fed fresh leaves cut from cherry trees provided *ad lib*. When a late 5^th^ instar caterpillar had purged its gut (caterpillars reached the late 5^th^ instar and gut purged between August 3^rd^, 2015 –August 13^th^, 2015), indicating the onset of cocoon spinning, the time of gut purge and the caterpillar’s post gut purge weight were recorded. Caterpillars were then immediately brought into the laboratory for cocoon spinning trials.

Trials were conducted in an isolated room, under controlled conditions (21°C, constant light conditions, 22% relative humidity) at the University of Massachusetts Medical School. As the construction of baggy cocoons in captivity has only been observed anecdotally, e.g., a few observed by amateur breeders, we developed an indoor cocoon spinning arena that lead to the reliable spinning of both cocoon-morphs, that consisted of a clear, plastic box (8.89 x 8.89 x 12.7 cm; US Acrylic), with a poplar dowel (length: 10.16 cm; diameter: 0.635 cm) positioned in the middle of the box to simulate a branch (Fig 6A). We placed paper grids (1 cm^2^ squares) on two arena walls perpendicular to each other, and on the bottom of the arena, to use for recording the 3D position (X, Y, and Z-coordinates) of the caterpillar’s head during a trial within the arena.

A cocoon spinning trial consisted of an 18-hour period, that started when the animal had settled on the dowel (time = 0) after being introduced into the arena, and had begun to add silk to the dowel and to parts of the arena, in order to start assembling its silk scaffold. Pilot trials demonstrated that at 18 hours, caterpillars had spun the full silhouette of an outer envelope, providing an outer envelope with an appropriate form for both identifying the cocoon-type being spun and for estimating its size using our grid system. After 18 hours, caterpillars had added a sufficient amount of silk to the walls of the outer envelope to occlude the caterpillar from view during cocoon construction and trials were terminated at this time. To standardize the cocoons examined in our trials, we only used the cocoons spun on the dowel itself in our behavioral analysis (e.g., the dowel provided the vertical axis of the cocoon, with the cocoon’s valve positioned at the top of the cocoon and on the dowel). Trials were continuously recorded for the full 18-hour period, using three cameras (Pro-550, Swann Communications), which were positioned at one of three viewing angles (XY, YZ, and XY planes of the arena). The footage from each camera was recorded using an eight channel H.264 DVR-8-2550 digital video recorder (Swann Communications), with each camera recorded onto a separate video channel. Due to the limited number of three-camera systems and recording channels at our disposal (two sets), we were unable to record all the caterpillars tested in our arenas during the entire testing period. Out of the 29 caterpillars we tested in our arenas, 18 caterpillars spun cocoons on the dowel (62% dowel rate); we were able to record the complete 18 hour spinning behavior of nine animals (4 baggy cocoons and 5 compact cocoons).

For the nine caterpillars that we recorded, we scored the spinning behavior of each animal at four hour intervals (1^st^, 4^th^, 8^th^, 12^th^, and 16^th^ hours of spinning) during the 18-hour period, using a behavioral repertoire expanded from that used by [5] (S1 Table). For each of these sampling hours for both cocoon-morphs, we calculated a mean time budget for each behavior performed during that hour, we constructed pooled common zero XYZ scatterplots (i.e., the same orientation within the arena for all cocoons) indicating where a behavior was done within the 3D space of the arena (stretch-bend (1–3 pulls), stretch-bend (>3 pulls), swing-swing, figure-8 during the scaffold stage were plotted in this manner; S1 Table), we produced a mean ethogram (e.g., [36]) to compare patterns of behavior between baggy and compact spinners, and we calculated the mean number of 180° and 360° body turns executed by caterpillars to compare the orientation of animals during cocoon construction. The size of each completed cocoon was estimated by calculating its volume using the arena grids. The time budgets for each of the behaviors across sampling hours, the frequency of 180° and 360° body turns in each hour, and cocoon size at 18 hours, were statistically compared between baggy and compact spinners using GraphPad Prism (Version 7.00, GraphPad Software, Inc.). For each sampling hour, we also compared the mean ethograms and pooled common zero XYZ scatterplots between baggy and compact spinners.

## Supporting information

S1 Appendix Time budgets of cocoon spinning behaviors. (DOCX)

S2 Appendix Ethograms analyzing cocoon spinning behavioral patterns. (DOCX)

S3 Appendix XYZ scatterplots of cocoon spinning behavior. (DOCX)

S4 Appendix Comparison of the thermoregulatory properties of dimorphic cecropia moth cocoons. (DOCX)

S1 Table Definition of behaviors used by *H*. *cecropia* silkworms during the silk scaffold and outer envelope construction stages. (DOCX)

S2 Table Time budget comparisons for the different construction behaviors used by *H*. *cecropia* silkworms to spin either a baggy or compact cocoon (silk scaffold and outer envelope stages), during the 18 hour cocoon construction period. (DOCX)

S3 Table Thermoregulatory performance of baggy and compact *H*. *cecropia* silkworm cocoons in heat-gain and heat-loss trials. (DOCX)

S1 Fig Mean ethograms for baggy and compact cocoon construction across all sampling periods. Arrow thickness indicates probability of a given behavior following another (0–1.0). Coding of behaviors: vertical motion during silk scaffold stage, SV; stretch-bend (1–3 pulls), SB3-; stretch-bend (>3 pulls), SB3+; horizontal motion during silk scaffold stage, SH; figure-8 motion during silk scaffold stage, S8; swing-swing, SS; manipulate silk scaffold, MS; vertical motion during outer envelope stage, V; horizontal motion during outer envelope stage, H; diagonal motion during outer envelope stage, D; manipulate outer envelope, MO; figure-8 motion during outer envelope stage, 8. For clarity, ethograms for the 12^th^ hour of trials for both baggy and compact spinners only show transition probabilities > 0.1.
(PDF)

S2 Fig Pooled common zero XYZ scatterplots in common arena cocoon spinning trials. Scatterplots show the location in the arena at which the three major cocoon spinning behaviors (stretch-bend, 1–3 and >3 pulls; swing-swing; figure-8 motion during silk scaffold stage) were performed by spinners of both cocoon-morphs, across all sampling periods. Each dot represents a single behavioral event. Baggy cocoon spinners denoted in blue and compact cocoon spinners denoted in red.
(PDF)

S1 Video 3D reconstructions of the relationship between the outer and inner envelopes of both baggy and compact cocoons. Purple denotes outer envelope; Green denotes inner envelope; baggy cocoon is the larger of the two cocoons.
(MP4)

S2 Video Cocoon construction behavior of *H*. *cecropia* silkworms assayed in our common spinning arena. (MP4)
