# Supplementary material for: Dimorphic cocoons of the cecropia moth (Hyalophora cecropia): Morphological, behavioral, and biophysical differences
Source: PLoS One. 2017 Mar 22;12(3):e0174023. doi: 10.1371/journal.pone.0174023 (PMC5362091; doi:10.1371/journal.pone.0174023)
Supplement: S1 Appendix — (DOCX) [file pone.0174023.s001.docx]

**S1 Appendix. Time budgets of cocoon spinning behaviors.**

Spinners of baggy cocoons spend more time performing behaviors which involve placing silk across a larger area of the spinning site (>3 pull stretch-bend, swing-swing, figure-8; Fig 7A-C; S2 Table) during the silk scaffold stage. In contrast, spinners of compact cocoons spend more time performing behaviors that involved adding silk in a more localized manner (vertical motion of adding silk to dowel; S2 Table). Compact spinners also manipulated the silk scaffold to a greater degree than baggy spinners (S2 Table). During the construction of the outer envelope, although baggy and compact spinners spend a similar amount of time filling in the walls of the outer envelope (to the point an animal is no longer in view), compact spinners spent a significantly greater amount of time constructing the valve of the outer envelope (adding silk in a vertical and diagonal motion to the valve; S2 Table).
