# Supplementary material for: Dimorphic cocoons of the cecropia moth (Hyalophora cecropia): Morphological, behavioral, and biophysical differences
Source: PLoS One. 2017 Mar 22;12(3):e0174023. doi: 10.1371/journal.pone.0174023 (PMC5362091; doi:10.1371/journal.pone.0174023)
Supplement: S2 Appendix — (DOCX) [file pone.0174023.s002.docx]

**S2 Appendix. Ethograms analyzing cocoon spinning behavioral patterns.**

Ethograms for the construction of both baggy and compact cocoons show that the sequences between construction behaviors are different between the cocoon-morphs, as demonstrated by differences in the transition probabilities between behaviors. These differences in patterns in cocoon construction behaviors between the cocoon-morphs were observed for all sampling periods (S1 Fig). Here, in conjunction with different time investments in the different behaviors (Fig 7, S1 Appendix, S2 Table), and different locations within the spinning arena for the performance of the different behaviors (S3 Appendix, S2 Fig), the spinning of either a baggy or compact cocoon depends on individuals using different patterns of construction behavior for the silk scaffold and outer envelope, throughout the 18 hour spinning period.
