# Supplementary material for: Dimorphic cocoons of the cecropia moth (Hyalophora cecropia): Morphological, behavioral, and biophysical differences
Source: PLoS One. 2017 Mar 22;12(3):e0174023. doi: 10.1371/journal.pone.0174023 (PMC5362091; doi:10.1371/journal.pone.0174023)
Supplement: S3 Appendix — (DOCX) [file pone.0174023.s003.docx]

**S3 Appendix. XYZ scatterplots of cocoon spinning behavior.**

When we compared the pooled common zero XYZ scatterplots for the major cocoon spinning behaviors between the two cocoon-morphs (S1 Table: stretch-bend, 1-3 pulls; stretch-bend, >3 pulls; swing-swing; figure-8 during silk scaffold stage), we found that the two cocoon-morphs differ in their locations of construction behavior as the 18 hour spinning period progresses (S2 Fig). These results demonstrate that caterpillars deposit silk at different locations within the common arena during the construction process, and along with different behavioral time budgets (Fig 7, S1 Appendix, and S2 Table) and patterning (S2 Appendix and S1 Fig), are the behavioral processes that underlie the construction of dimorphic cocoons.
