# Supplementary material for: Dimorphic cocoons of the cecropia moth (Hyalophora cecropia): Morphological, behavioral, and biophysical differences
Source: PLoS One. 2017 Mar 22;12(3):e0174023. doi: 10.1371/journal.pone.0174023 (PMC5362091; doi:10.1371/journal.pone.0174023)
Supplement: S4 Appendix — (DOCX) [file pone.0174023.s004.docx]

**S4 Appendix. Comparison of the thermoregulatory properties of dimorphic cecropia moth cocoons.**

In thermoregulation convection trials, intact baggy cocoons acted as greater heat sinks than compact cocoons in heat-gain trials (S3 Table). Baggy cocoons had an internal temperature that started to increase faster (shorter heat gain lag time) and reached a higher maximum temperature, with the inner temperature hotter for a longer portion of the trial (greater area under the curve; AUC), than that of compact cocoons. Although there was no difference in the actual final temperature reached at the end of the trial period between the two cocoon-morphs, the predicted final temperature for baggy cocoons was greater than that of compact cocoons. These differences were only seen when cocoons were intact, as no difference in heat-gain temperature parameters was seen between the two cocoon-morphs when the outer envelope and silk from the intermediate space were absent during trials. We found no difference in the heating rate between the cocoon-morphs, as there was no difference in k constants between baggy and compact cocoons, with or without the presence of the outer envelope and intermediate space silk. In contrast, for thermoregulation convection trials in which heat-loss was measured, we found no differences between the cocoon-morphs in thermoregulatory performance (S3 Table).

When we used infrared (IR) radiation as a heat source for thermoregulation trials, we found a similar pattern of differences in thermoregulatory performance in heat-gain trials between the cocoon-morphs as seen in convection trials (S3 Table). Here, however, certain differences between baggy and compact cocoons were greater in magnitude as compared to what was observed in heat-gain convection trials. These included the duration that a cocoon had heat during trials, the maximum and final temperatures at the end of trials, and the predicted final temperatures. In contrast to heat-loss convection trials that showed no difference between the cocoon-morphs, heat-loss trials involving IR heat showed that baggy cocoons can retain more heat during the trial period, and have a higher predicted final temperature, than compact cocoons. For both IR heat-gain and heat-loss trials, the differences between the cocoon-morphs only occurred when cocoons were intact (S3 Table).

Because baggy cocoons act as greater heat sinks when heated with IR light, we further examined the role of the outer envelope, and intermediate space silk, in this IR heating effect. In trials that compared the initial thermal performance of intact baggy cocoons with a subsequent trial in which these cocoons had no outer envelope, but still had the intermediate space silk attached to the inner envelope (Fig 8A), we found no difference in heating performance between both conditions (final trial temperature normalized to whole cocoon conditions; Paired t-test: *t*_2_ = 0.402, *P* > 0.7, *N* = 3; Fig 8B). In a following set of trials, we found that baggy cocoons with intermediate space silk attached to the inner envelope had greater final trial temperatures than in trials in which the intermediate space silk was removed (i.e., inner envelope alone) (Paired t-test: *t*_2_ = 4.95, *P* = 0.0385, *N* = 3; Fig 8C). Taken together, these results show that the enhanced IR heating effect found in baggy cocoons requires the silk found in the intermediate space of baggy cocoons (Fig 8D); the difference in IR heating between the cocoon-morphs is due to baggy cocoons having significantly more silk in the intermediate space for IR radiation to act on than compact cocoons. The outer envelope has a passive role in IR heating only, but despite this, the outer envelope of baggy cocoons plays an important role to facilitate heating as it allows for more IR radiation to be transmitted into the cocoon relative to the outer envelope of compact cocoons (Fig 8E). Of the four types of cocoons envelopes (outer envelope and inner envelope for both cocoon-morphs, *N* = 10 for all groups; Fig 8F), we found that baggy outer envelopes allow the greatest percent transmission of IR wavelengths (Kruskal-Wallis test: p < 0.0001; *P* < 0.01 for all comparisons between baggy outer envelope and all other envelope types; Fig 8F), with the three other types having similar IR transmission (*P* > 0.9 for all comparisons; Fig 8F). This makes intact baggy cocoons have a greater percent transmission index for IR wavelengths into the interior of the cocoon, than intact compact cocoons (Unpaired t-test: *t*_11.30_ = 3.979, *P* = 0.002, *N* = 10 for both groups; Fig 8G).
