# Supplementary material for: Dimorphic cocoons of the cecropia moth (Hyalophora cecropia): Morphological, behavioral, and biophysical differences
Source: PLoS One. 2017 Mar 22;12(3):e0174023. doi: 10.1371/journal.pone.0174023 (PMC5362091; doi:10.1371/journal.pone.0174023)
Supplement: S1 Table — (DOCX) [file pone.0174023.s005.docx]

**S1 Table. Definition of behaviors used by *H. cecropia* silkworms during the silk scaffold and outer envelope construction stages.**

| **Stage^*^** | **Behavior** | **Description of how silk is attached or added^#^** |
| --- | --- | --- |
| SSc | Stretch-bend  (1-3 pulls) | Attach silk via contact at one location, and using 1-3 distinct pulling motions, extrude silk thread and bend body to attach silk to a second location |
|  | Stretch-bend  (>3 pulls) | Attach silk via contact at one location, and using >3 distinct pulling motions, extrude silk thread and bend body to attach silk to a second location |
|  | Swing-swing | Add silk to surface using back-and-forth swinging motion, while in continuous contact with the surface |
|  | Figure-8 | Add silk to surface using figure-8 motion, while in continuous contact with the surface |
|  | Vertical motion | Add silk to surface using vertical motion (head and spinneret movement perpendicular to arena floor) |
|  | Horizontal motion | Add silk to surface using horizontal motion (head and spinneret movement parallel to arena floor) |
|  | Manipulate | Manipulate SSc by adding silk to threads, or pulling SSc threads using legs or mandibles |
| OE | Figure-8 | Add silk to walls of OE using figure-8 motion |
|  | Vertical motion | Add silk to walls or valve of OE using vertical motion |
|  | Horizontal motion | Add silk to walls or valve of OE using horizontal motion |
|  | Diagonal motion | Add silk to walls or valve of OE using diagonal motion |
|  | Manipulate | Manipulate walls or valve of OE by pulling with either the legs or mandibles |

^*^Stage of cocoon construction; SSc: silk scaffold; OE: outer envelope layer

^#^During the attachment or addition of silk in the spinning arena, caterpillars extrude silk from their spinneret appendage, found at the base of the caterpillar’s face, below the mandibles.
