# Supplementary material for: Dimorphic cocoons of the cecropia moth (Hyalophora cecropia): Morphological, behavioral, and biophysical differences
Source: PLoS One. 2017 Mar 22;12(3):e0174023. doi: 10.1371/journal.pone.0174023 (PMC5362091; doi:10.1371/journal.pone.0174023)
Supplement: S2 Table — (DOCX) [file pone.0174023.s006.docx]

**S2 Table. Time budget comparisons for the different construction behaviors used by *H. cecropia* silkworms to spin either a baggy or compact cocoon (silk scaffold and outer envelope stages), during the 18 hour cocoon construction period.**

| **Stage** | **Behavior** | **Test statistics^a^** | **Variable effects** | **Relationship between baggy and compact spinners^b^** |
| --- | --- | --- | --- | --- |
| SS and OE | Stretch-bend (1-3 pulls) | F_(4,28)_=3.494, P=0.0196 | Interaction | Compact spinners perform behavior more during the 4^th^ hour (P=0.0212) than baggy spinners. |
| SS and OE | Stretch-bend (>3 pulls) | F_(4,28)_=4.137, P=0.0093 | Interaction | Baggy spinners perform behavior more during the 4^th^ hour (P=0.0073) than compact spinners. |
| SS and OE | Swing-swing | F_(4,28)_=4.023, P=0.0106 | Interaction | Baggy spinners perform behavior more than compact spinners during the 1^st^ (P=0.0169) and 8^th^ hours (P=0.0085). |
| SS | Figure-8 | F_(4,28)_=3.56, P=0.0181 | Interaction | Baggy spinners have consistent behavior until significant decrease during the 16^th^ hour (P<0.05 for comparisons between 16^th^ hour and other sampling periods). Compact spinners have high (1^st^ and 8^th^) and low (4^th^, 12^th^, and 16^th^) performance of behaviors, with high and low time periods different from each other (P<0.05). |
| SS | Vertical motion | F_(4,28)_=5.583, P=0.002 | Interaction | Compact spinners spend more time than baggy spinners during the 1^st^ hour (P=0.0005). |
| SS | Horizontal motion | F_(4,28)_=0.7537, P>0.5 | None | No difference between spinners; no interaction effect and no main effects (cocoon-morph, P>0.4; time, P>0.3) |
| SS | Manipulate SS | F_(4,28)_=1.398, P>0.2 | Main | Compact spinners manipulate SS more than baggy spinner throughout the trial (main effect of cocoon-morph, P=0.0064). |
| OE | Figure-8 | F_(4,28)_=0.1502, P>0.9 | None | Spinners of both morphs spend a similar amount of time filling in the walls of the OE; no interaction effect and no main effects (e.g., morph, P>0.6). |
| OE | Vertical motion | F_(4,28)_=3.894, P=0.0123 | Interaction | Compact spinners spend more time during the 12^th^ hour (P=0.0005). |
| OE | Horizontal motion | F_(4,28)_=0.0785, P>0.9 | Main | No difference between spinners, no difference between morphs (P>0.7), but both groups only perform behavior during the 12^th^ hour (main effect of time, P=0.0008). |
| OE | Diagonal motion | F_(4,28)_=9.513, P<0.0001 | Interaction | Compact spinners spend more time than baggy spinners during the 12^th^ (P<0.0001) and 16^th^ (P=0.0012) hours. |
| OE | Manipulate OE | F_(4,28)_=0.8797, P>0.4 | None | No difference between morphs; no interaction effect and no main effects (e.g., morph, P>0.2). |

^a^Two-way repeated measures ANOVA was used to statistically compare baggy (N=4) and compact (N=5) spinners for the time budget for each behavior scored, with cocoon-morph (baggy or compact) and sampling period (behaviors sampled at the 1^st^, 4^th^, 8^th^, 12^th^, and 16^th^ hours of trials; repeated measure) as variables.

^b^We only report variables with significant interaction or main effects, and post-hoc comparisons between the different cocoon-morphs in which there was a significant difference.

SS=silk scaffold; OE=outer envelope layer
