# Supplementary material for: Dimorphic cocoons of the cecropia moth (Hyalophora cecropia): Morphological, behavioral, and biophysical differences
Source: PLoS One. 2017 Mar 22;12(3):e0174023. doi: 10.1371/journal.pone.0174023 (PMC5362091; doi:10.1371/journal.pone.0174023)
Supplement: S3 Table — (DOCX) [file pone.0174023.s007.docx]

**S3 Table. Thermoregulatory performance of baggy and compact *H. cecropia* silkworm cocoons in heat-gain and heat-loss trials.**

| **Trial** | **Performance** | **Heat source** | **Test statistics** | **Comparisons within a cocoon-morph** | **Comparisons between cocoon-morphs** |
| --- | --- | --- | --- | --- | --- |
| Gain | Lag time (s) | Convection | F_(1,10)_=21.20, P=0.001^a^ | - No difference with or without OE for baggy (P>0.1) - Compact starts heating faster with OE (P=0.0021) | - Whole cocoons: baggy starts heating faster than whole compact (P=0.0276) - IE only: no difference between morphs (P>0.9) |
|  |  | IR | F_(1,10)_=15.86, P=0.0026^a^ | - No difference with or without OE for baggy (P>0.1) - Compact starts heating faster with OE (P=0.0086) | - No difference between morphs as whole cocoons (P>0.2) or with IE only (P>0.9) |
|  | Cocoon interior hotter than exterior during trial (AUC) | Convection | F_(1,10)_=10.61, P=0.0086^a^ | - No difference with or without OE for either baggy (P>0.7) or compact (P>0.2) cocoons | - Whole cocoons: baggy spend more time with interior heat than compact (P=0.0135) - IE only: no difference between morphs (P>0.8) |
|  |  | IR | F_(1,10)_=10.61, P=0.0086^a^ | - Baggy have more heat as whole cocoons (P=0.0008) - No difference with or without OE for compact (P>0.7) | - Whole cocoons: baggy spend more time with interior heat than compact (P=0.0003) - IE only: no difference between morphs (P>0.9) |
|  | Maximum temperature reached (Cocoon-Control; °C) | Convection | F_(1,10)_=9.055, P=0.0131^a^ | - No difference with or without OE for either baggy (P>0.6) or compact (P>0.1) cocoons | - Whole cocoons: baggy reach higher max temperature than compact (P=0.0053) - IE only: no difference between morphs (P>0.9) |
|  |  | IR | F_(1,10)_=8.443, P=0.0157^a^ | - Baggy reach higher max temperature with OE than without (P=0.0011) - No difference with or without OE for compact (P>0.6) | - Whole cocoons: baggy reach higher max temperature than compact (P=0.0003) - IE only: no difference between morphs (P>0.8) |
|  | Temperature at 10 minutes of trials (Cocoon-Control; °C) | Convection | F_(1,10)_=11.41, P=0.007^a^ | - Baggy hotter after 10 minutes with OE (P=0.0434) - No difference after 10 minutes with or without OE for compact (P>0.1) | - No difference between morphs with (P>0.3) or without (P>0.6) OE |
|  |  | IR | F_(1,10)_=11.66, P=0.0066^a^ | - Baggy hotter after 10 minutes with OE (P=0.0001) - No difference after 10 minutes with or without OE for compact (P>0.2) | - Whole cocoons: baggy has higher temperature after 10 minutes than compact (P=0.0004) - IE only: no difference between morphs (P>0.9) |
|  | Heating rate (estimated k constants) | Convection | F_(1,10)_=1.338, P>0.2^a^ | - No difference with or without OE for both baggy and compact | - No difference between baggy or compact with or without OE |
|  |  | IR | F_(1,10)_=64.33, P<0.0001^a^ | - Presence of OE makes both baggy (P=0.0002) and compact (P=0.001) heat faster | - No difference between baggy and compact as whole cocoon (P>0.8) or with IE alone (P>0.8) |
|  | Predicted final temperature (Cocoon-Control; °C) | Convection | F_(1,10)_=8.766, P=0.0143^a^ | - No difference with or without OE for both baggy (P>0.7) and compact (P>0.1) | - Whole cocoons: baggy has higher predicted final temperature than compact (P=0.0053) - IE only: no difference between morphs (P>0.9) |
|  |  | IR | F_(1,10)_=11.74, P=0.0065^a^ | - Baggy has higher predicted final temperature with OE than without (P=0.0002) - No difference with or without OE for compact (P>0.3) | - Whole cocoons: baggy has higher predicted final temperature than compact (P=0.0001) - IE only: no difference between morphs (P>0.9) |
| Loss | Lag time (s) | Convection | *t*_(7)_=2.264, P=0.06^b^ | n/a | - Whole cocoons: no difference between morphs |
|  |  | IR | F_(1,10)_=0.094, P>0.7^a^ | - No difference with or without OE for both baggy and compact | - No difference between baggy and compact with or without OE |
|  | Cocoon interior hotter than exterior during trial (AUC) | Convection | P=0.07^c^ | n/a | - Whole cocoons: no difference between morphs |
|  |  | IR | F_(1,10)_=10.69, P=0.0084^a^ | - Both baggy (P<0.0001) and compact (P=0.0002) cocoons retain heat for a longer period with OE | - Whole cocoons: baggy retain heat for a longer period than compact (P=0.0009) - IE only: no difference between morphs (P>0.7) |
|  | Temperature at 10 minutes of trials (Cocoon-Control; °C) | Convection | *t*_(9.747)_=1.885, P=0.09^b^ | n/a | - Whole cocoons: no difference between morphs |
|  |  | IR | F_(1,10)_=22.42, P=0.0008^a^ | - Baggy: higher temperature with OE than without (P=0.0040) - Compact: temperature the same with or without OE (P=0.06) | - No difference between baggy and compact with (P>0.2) or without (P>0.9) OE |
|  | Cooling rate (estimated k constants) | Convection | *t*_(7.816)_=1.692, P>0.1^b^ | n/a | - Whole cocoons: no difference between morphs |
|  |  | IR | F_(1,10)_=17.42, P=0.0019^a^ | - Both baggy (P=0.0259) and compact (P=0.032) cocoons cool slower with OE | - No difference between baggy and compact with (P>0.5) or without (P>0.5) OE |
|  | Predicted final temperature (Cocoon-Control; °C) | Convection | P>0.4^c^ | n/a | - Whole cocoons: no difference between morphs |
|  |  | IR | F_(1,10)_=68.11, P<0.0001^a^ | - Both baggy (P<0.0001) and compact (P=0.0027) have higher final temperature with OE than without | - Whole cocoons: baggy maintain higher temperature than compact (P=0.0134) - IE only: no difference between morphs (p>0.9) |

^a^We used mixed-model two-way ANOVA (variables: baggy or compact cocoon morph; whole cocoon or inner envelope only as repeated measure) to compare the different cocoon envelopes (baggy and compact outer envelope; baggy and compact inner envelope) in the different measures of thermoregulatory performance, and report Sidak’s multiple comparisons P values for post-hoc comparisons tests. For heat-loss convection trials, we compared whole baggy and whole compact cocoons only, and statistically analyzed data using either a t-test^b^ or Mann-Whitney^c^ test. N=6 for each type of envelope for both baggy and compact cocoons; AUC = area under temperature curve; OE = outer envelope; IE = inner envelope; IR = infrared.
