# Supplementary material for: Dimorphic cocoons of the cecropia moth (Hyalophora cecropia): Morphological, behavioral, and biophysical differences
Source: PLoS One. 2017 Mar 22;12(3):e0174023. doi: 10.1371/journal.pone.0174023 (PMC5362091; doi:10.1371/journal.pone.0174023)
Supplement: S1 Fig — Arrow thickness indicates probability of a given behavior following another (0–1.0). Coding of behaviors: vertical motion during silk scaffold stage, SV; stretch-bend (1–3 pulls), SB3-; stretch-bend (>3 pulls), SB3+; horizontal motion during silk scaffold stage, SH; figure-8 motion during silk scaffold stage, S8; swing-swing, SS; manipulate silk scaffold, MS; vertical motion during outer envelope stage, V; horizontal motion during outer envelope stage, H; diagonal motion during outer envelope stage, D; manipulate outer envelope, MO; figure-8 motion during outer envelope stage, 8. For clarity, ethograms for the 12th hour of trials for both baggy and compact spinners only show transition probabilities > 0.1. (PDF) [file pone.0174023.s008.pdf]

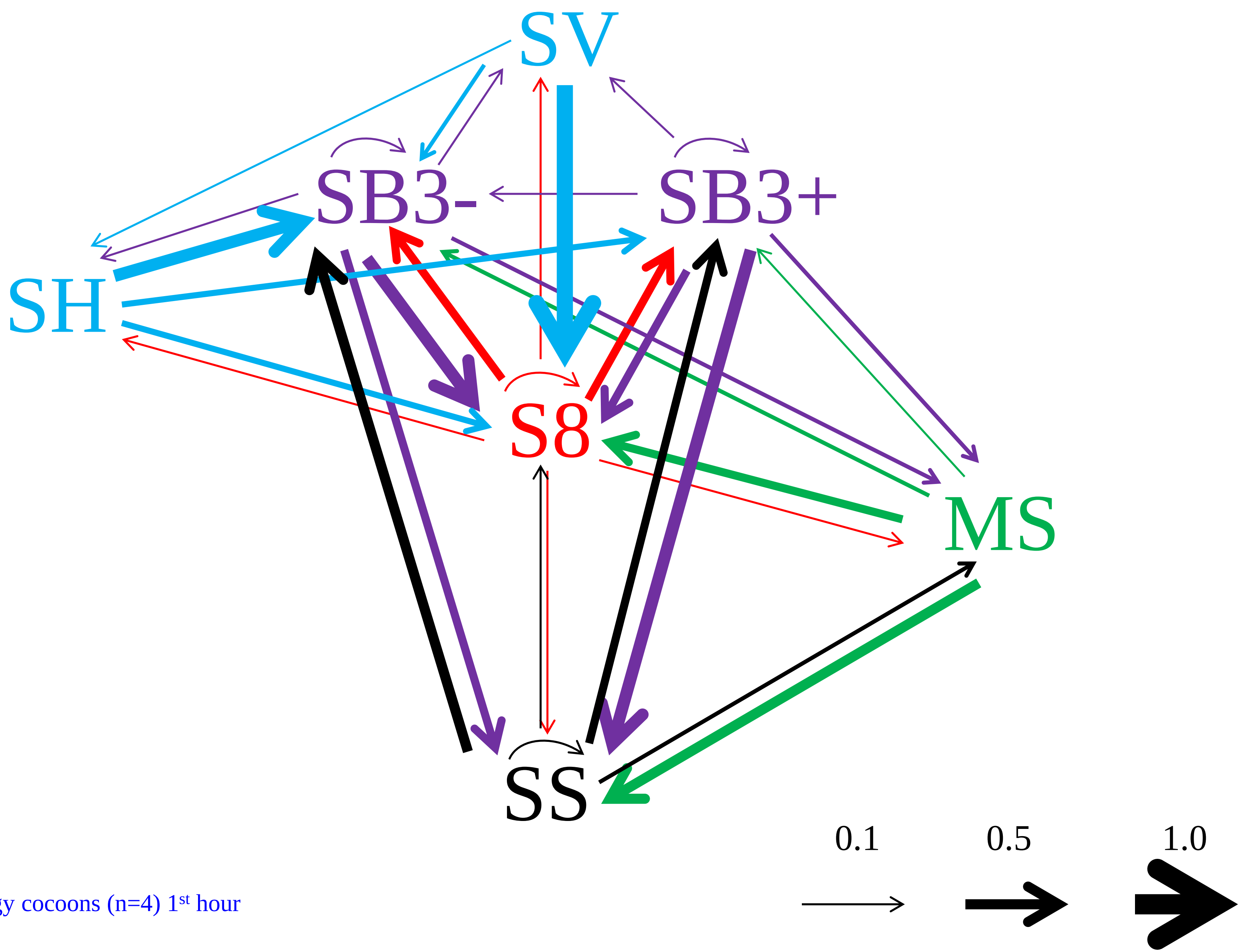

Ethogram: baggy cocoons (n=4) 1st hour

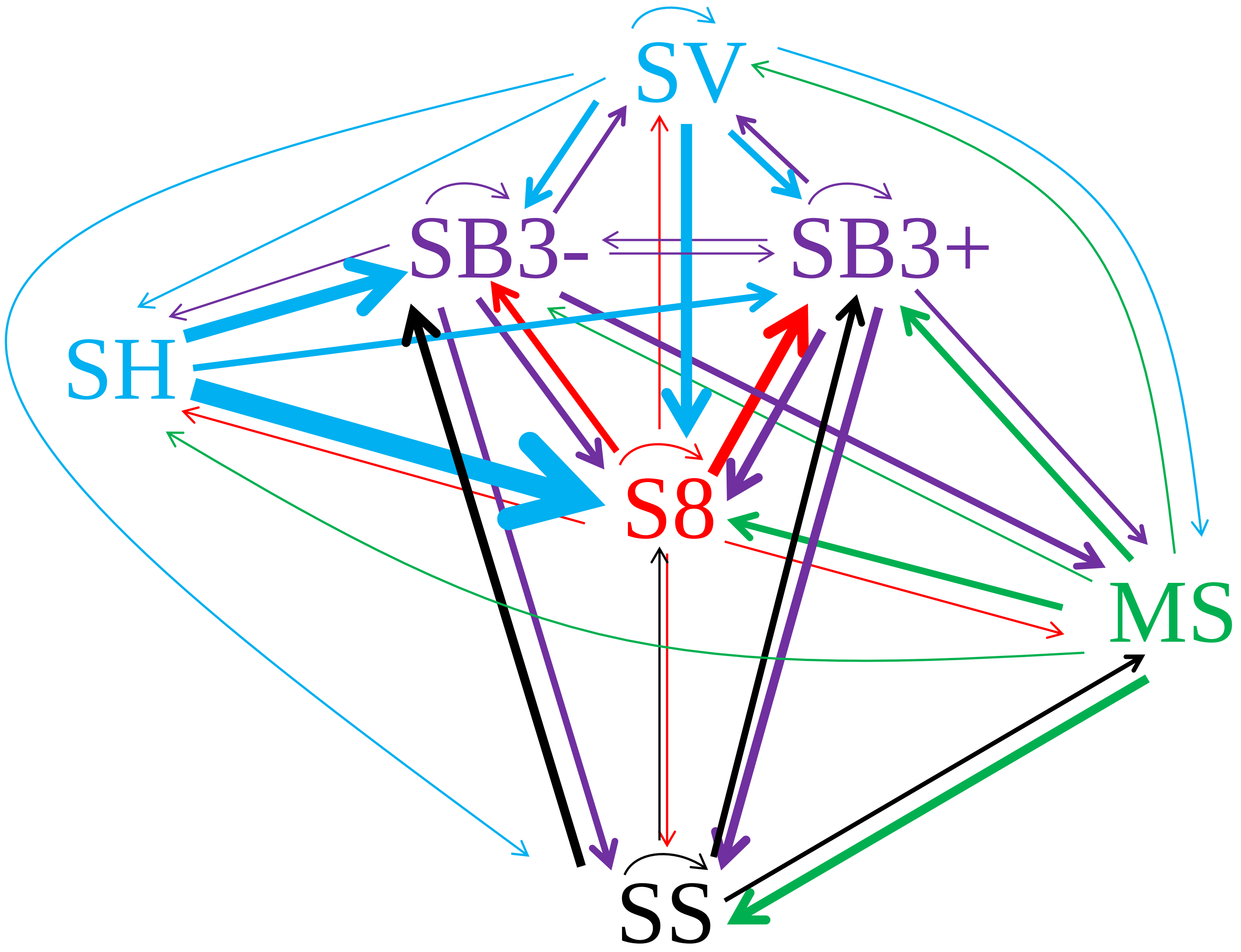

Ethogram: compact cocoons (n=5) 1st hour

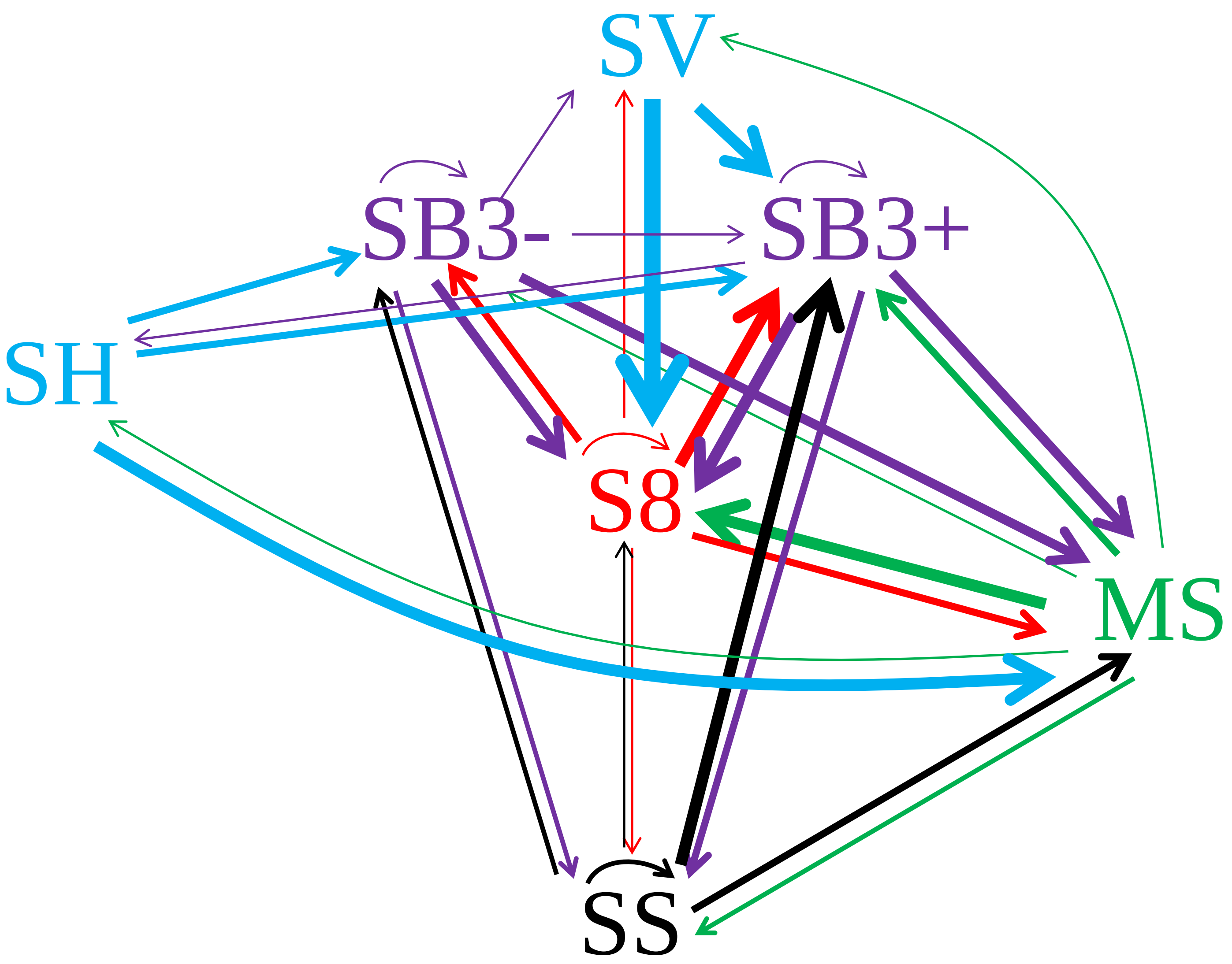

Ethogram: baggy cocoons (n=4) 4th hour

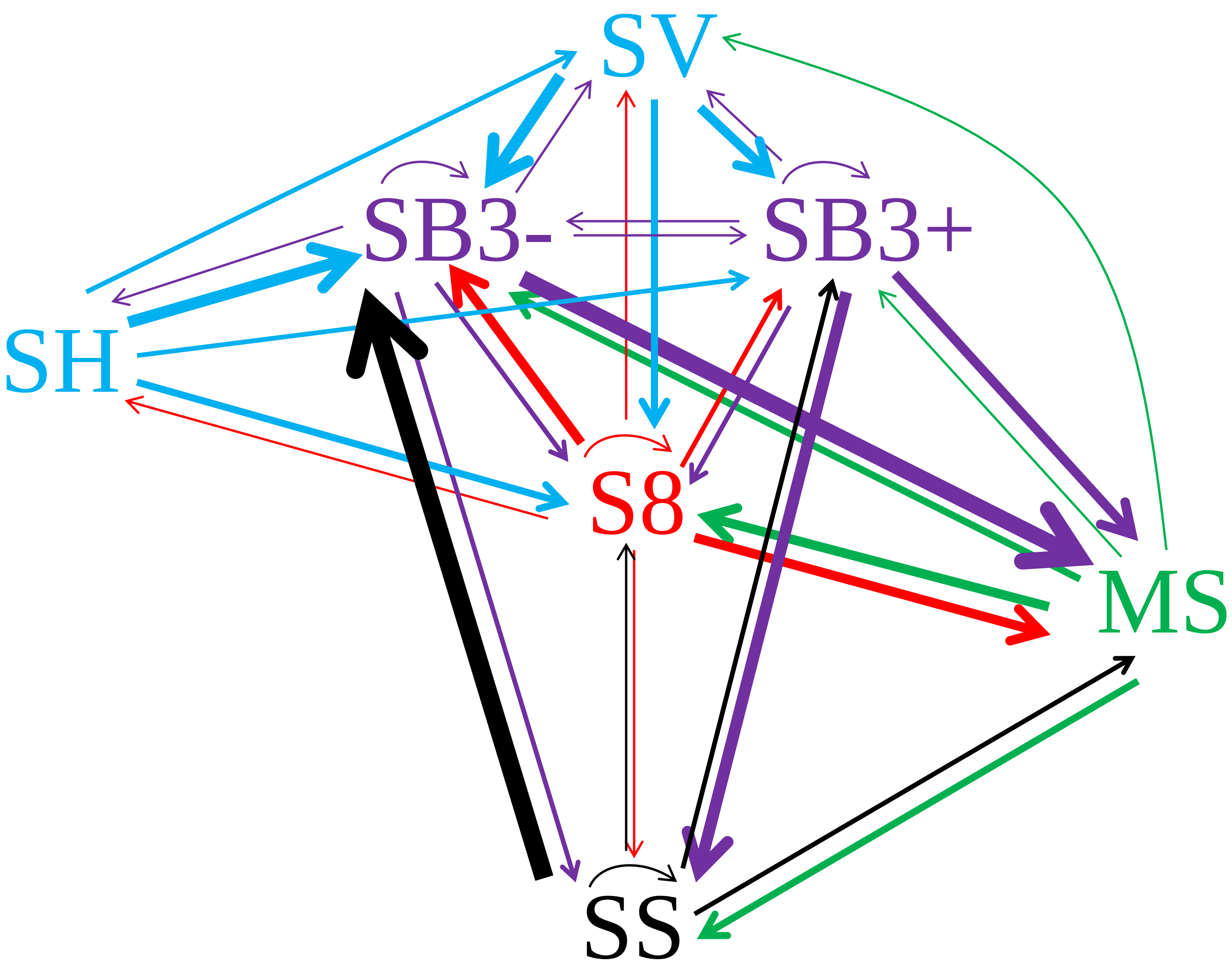

Ethogram: compact cocoons (n=5) 4th hour

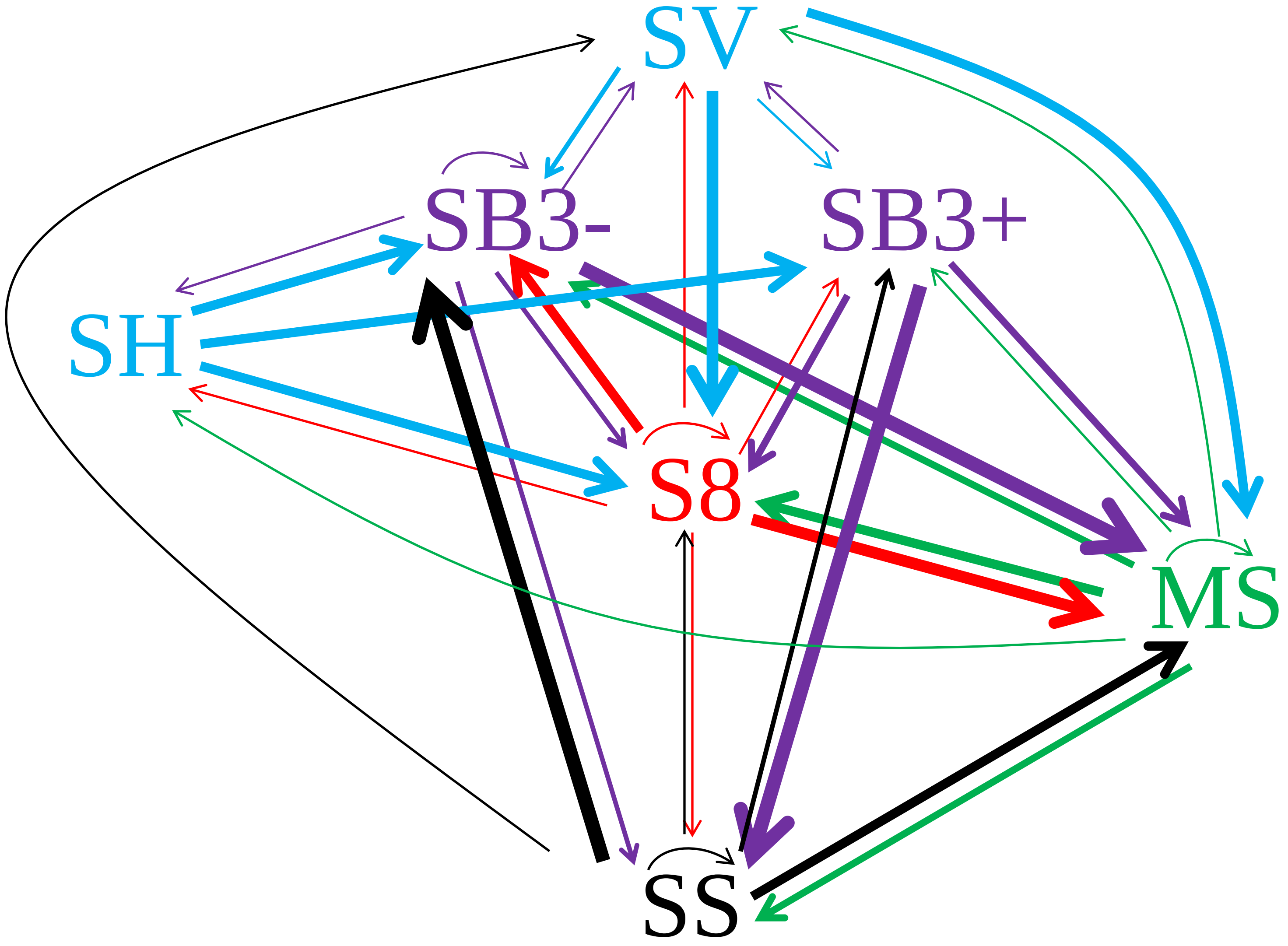

Ethogram: baggy cocoons (n=4) 8th hour

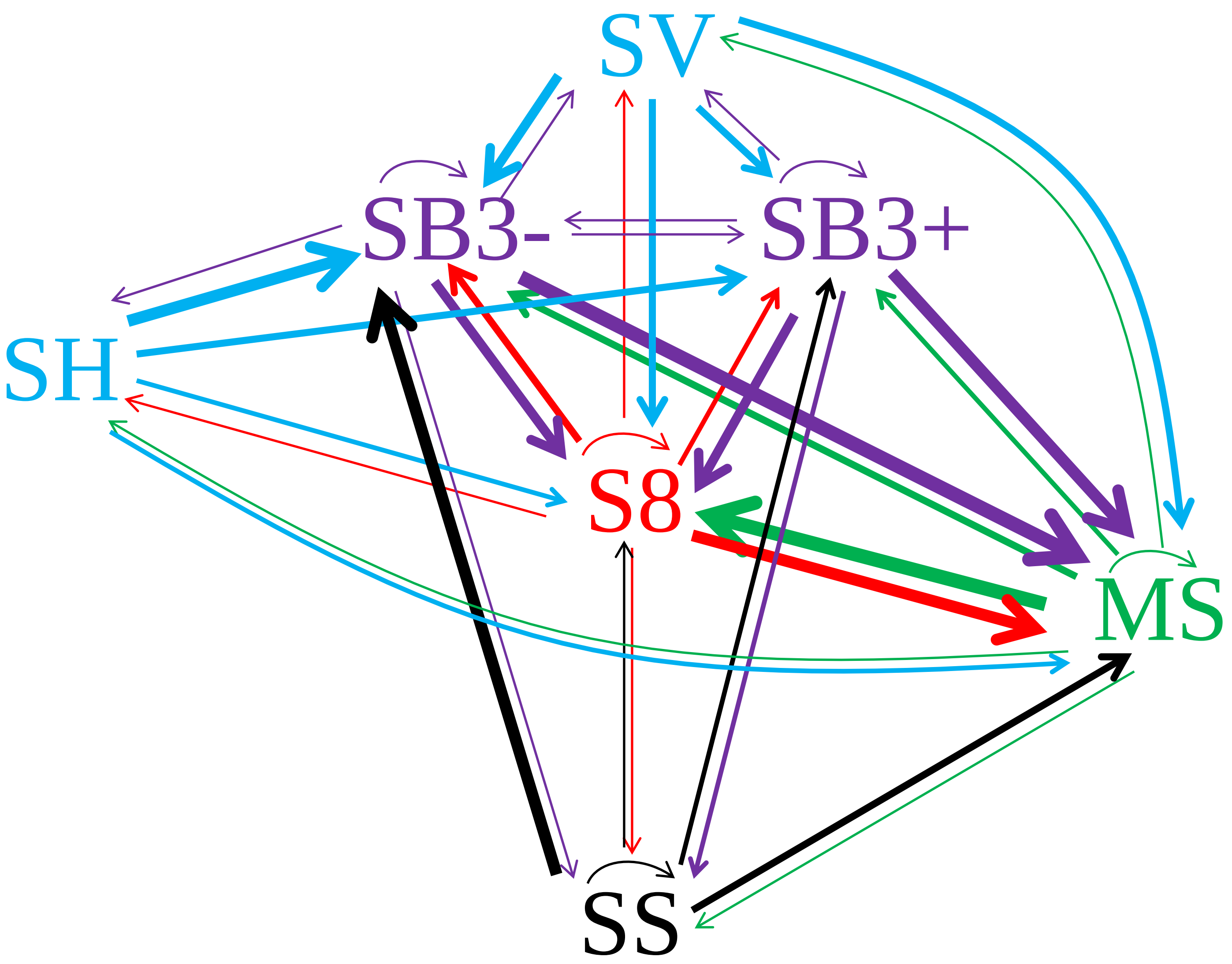

Ethogram: compact cocoons (n=5) 8th hour

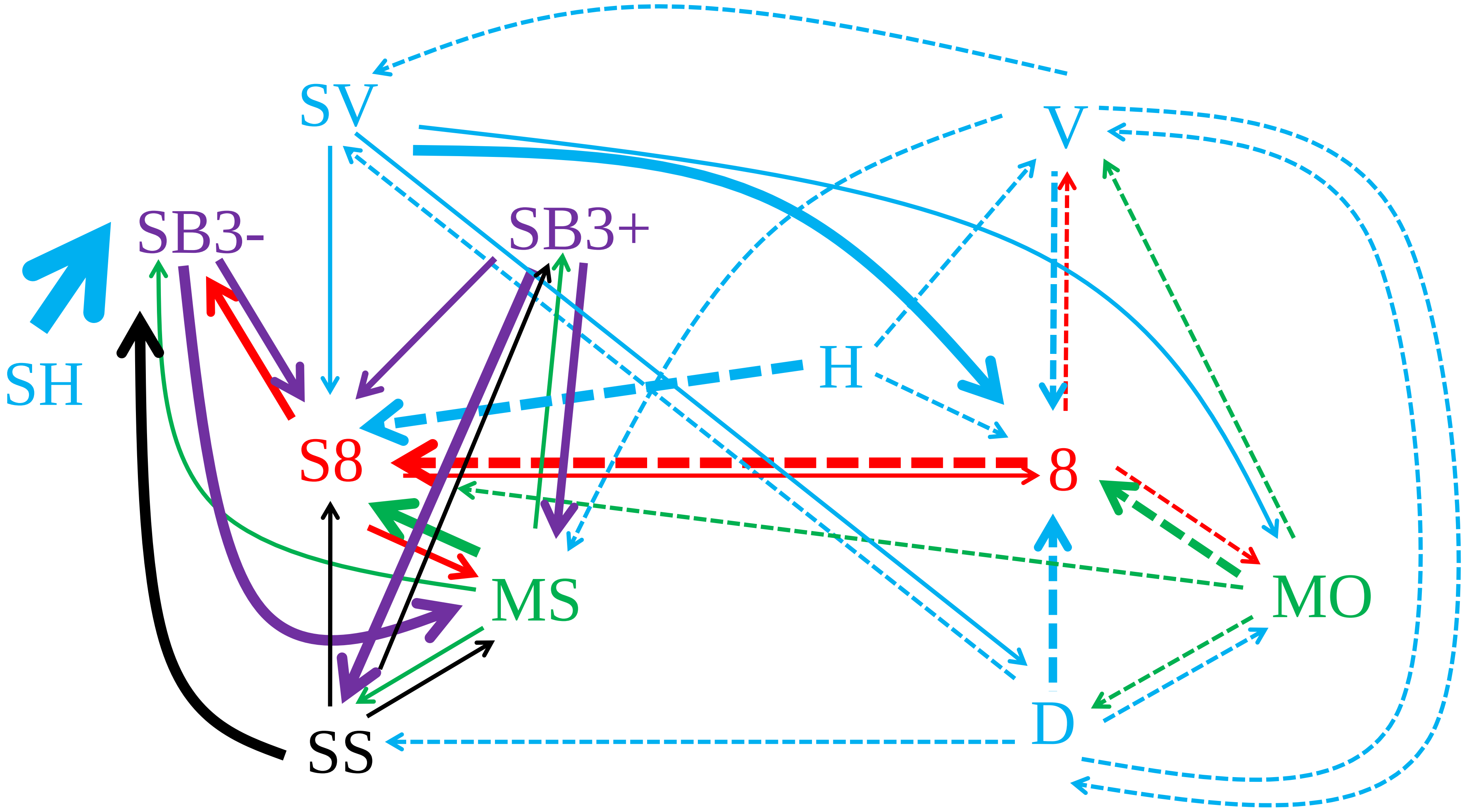

Ethogram: baggy cocoons (n=4) 12th hour, transitions >0.10 only

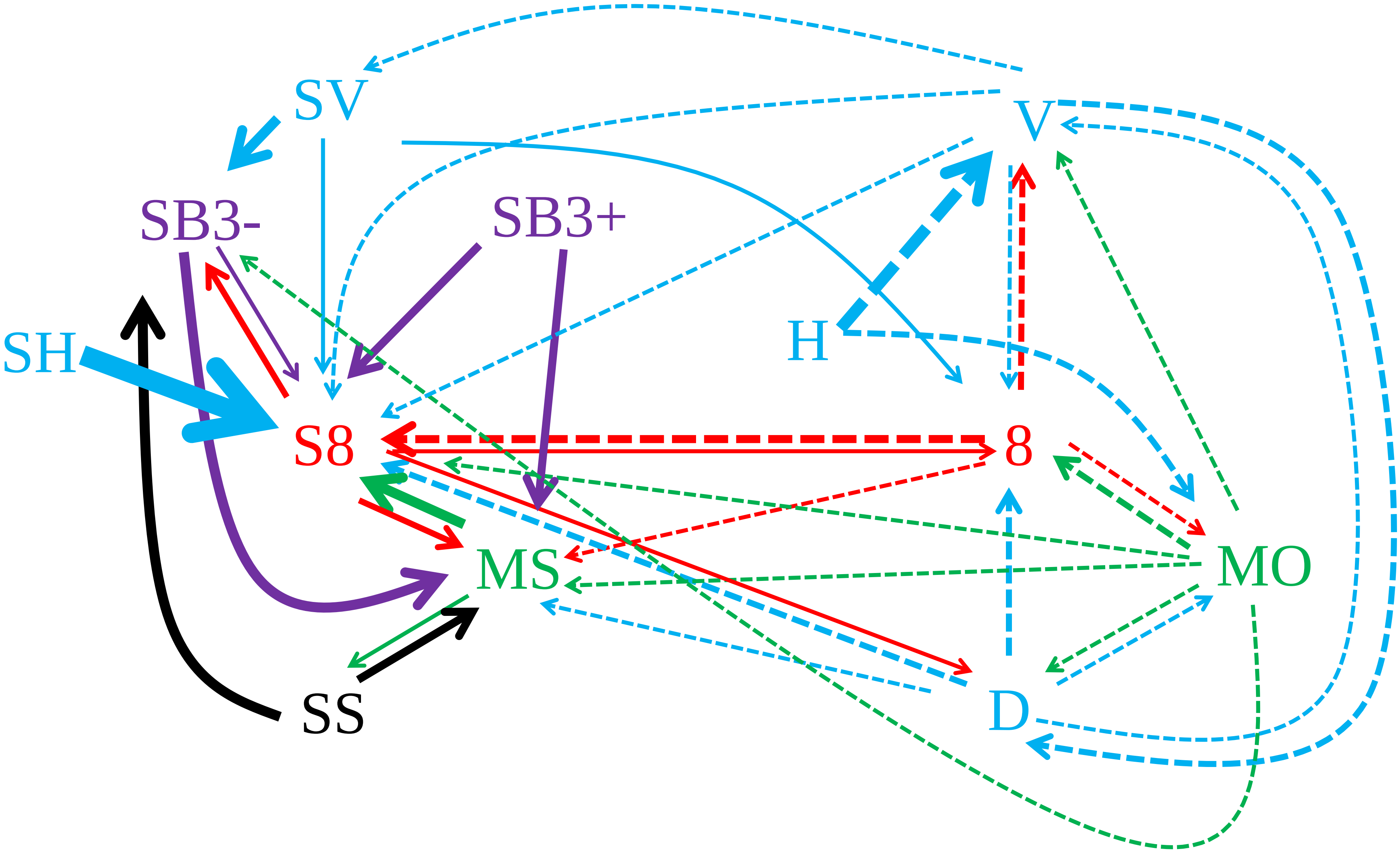

Ethogram: compact cocoons (n=5) 12th hour, transitions >0.10 only

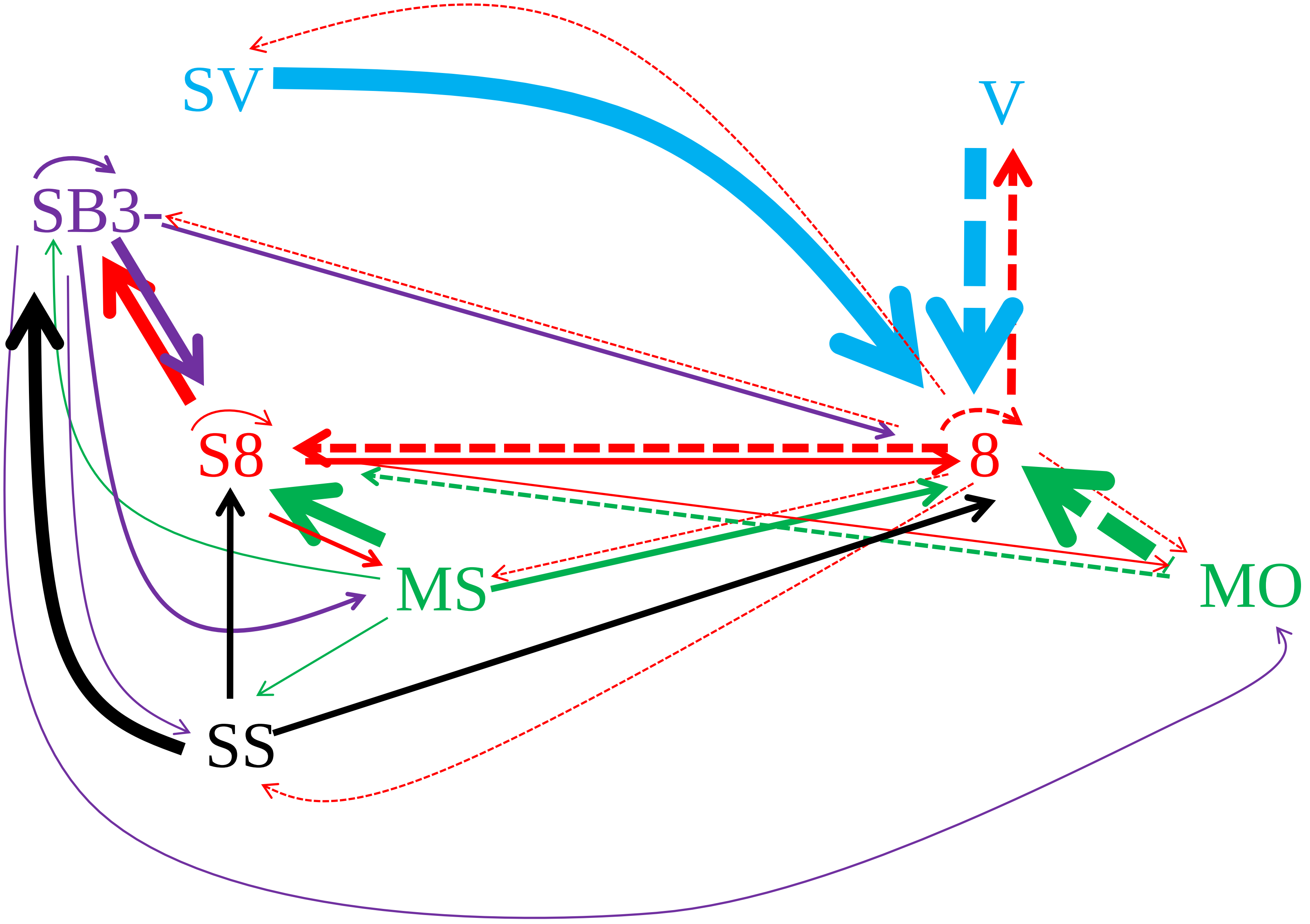

Ethogram: baggy cocoons (n=4) 16th hour

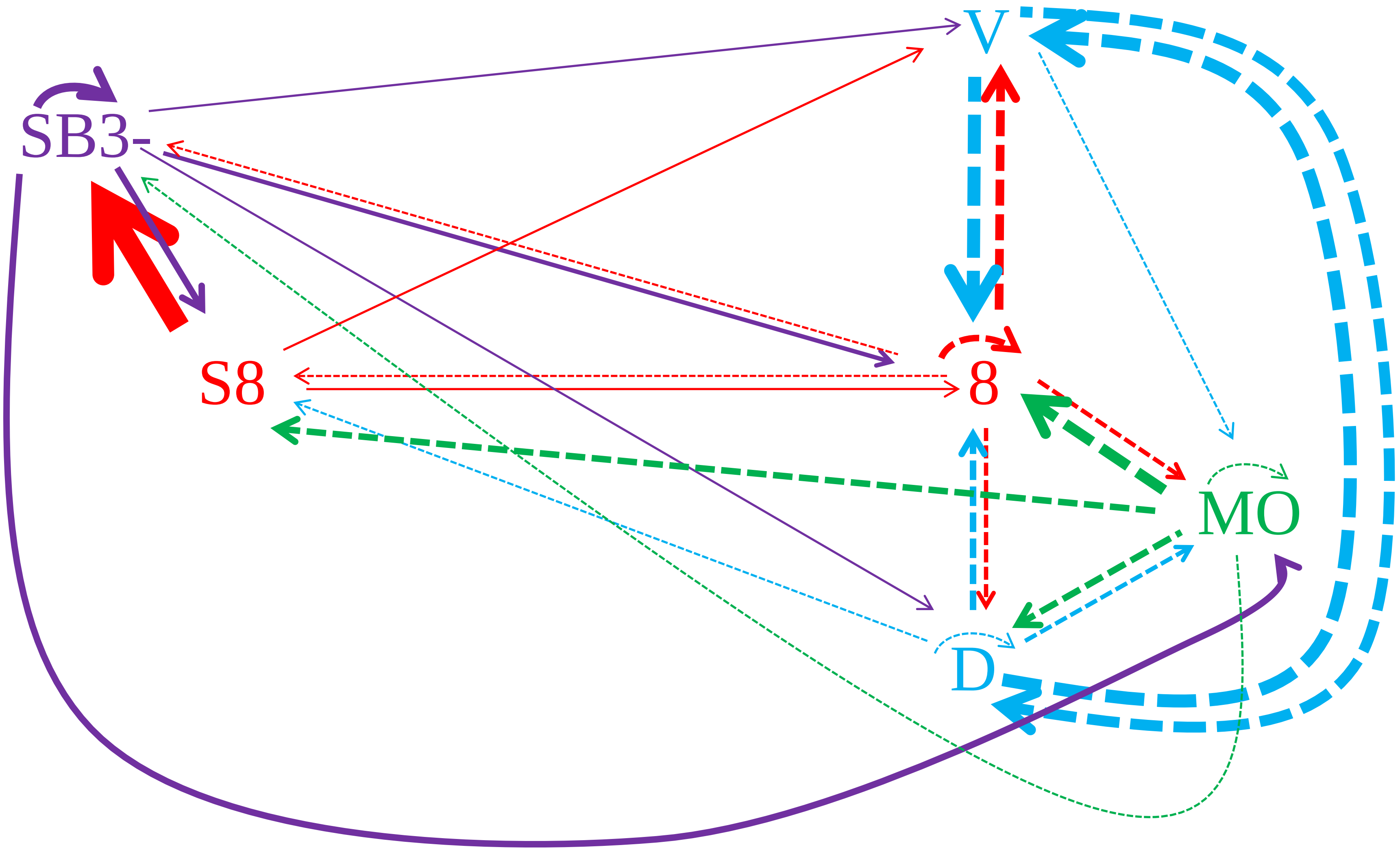

Ethogram: compact cocoons (n=5) 16th hour
