# Supplementary material for: Dimorphic cocoons of the cecropia moth (Hyalophora cecropia): Morphological, behavioral, and biophysical differences
Source: PLoS One. 2017 Mar 22;12(3):e0174023. doi: 10.1371/journal.pone.0174023 (PMC5362091; doi:10.1371/journal.pone.0174023)

Stretch-bend (1-3 pulls) (1st hour)

Baggy (N=4)

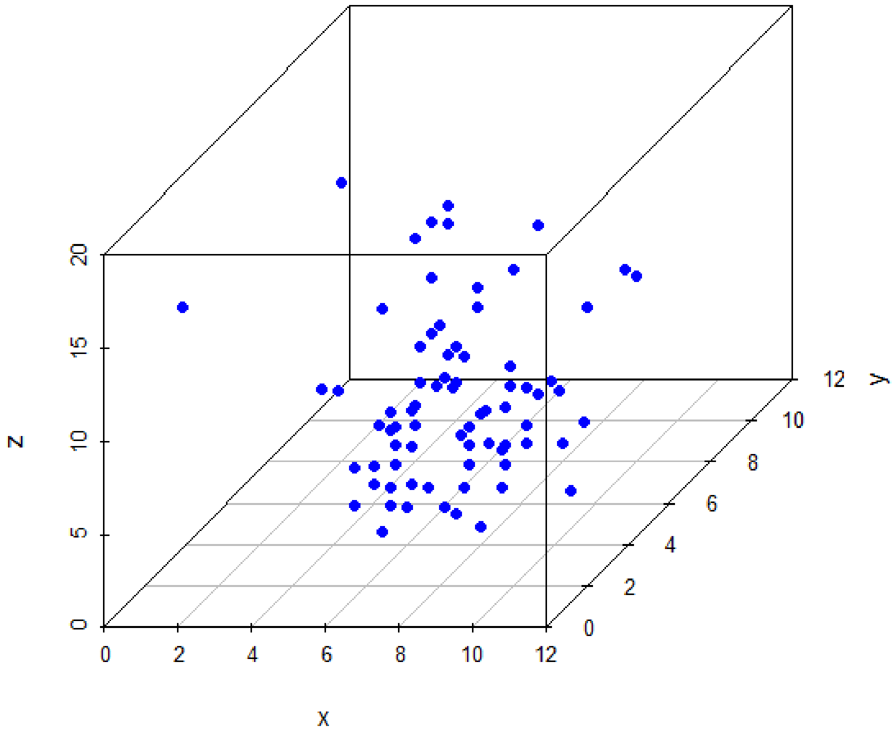

Compact (N=5)

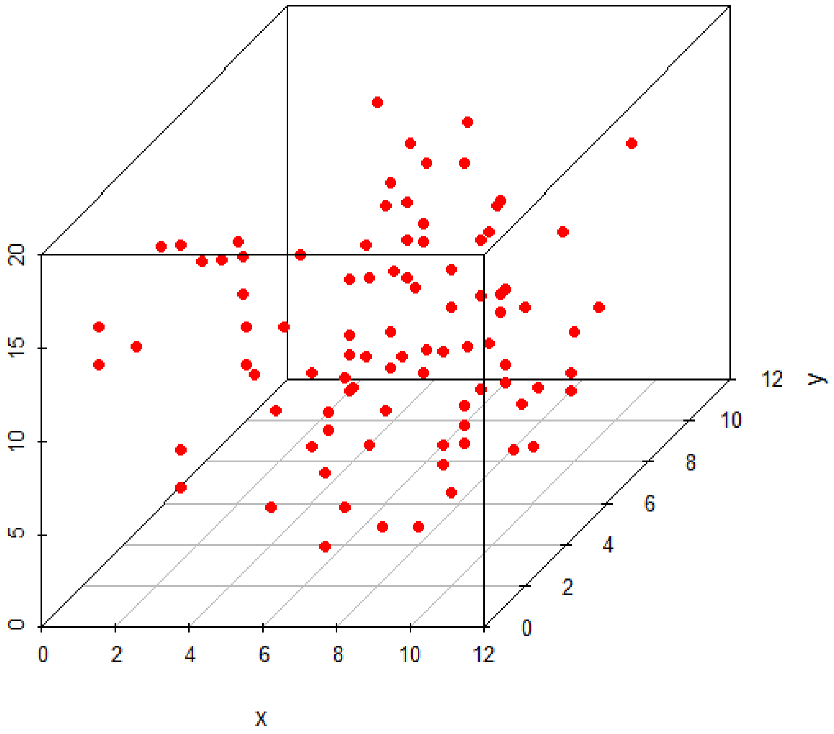

Stretch-bend (1-3 pulls) (4th hour)

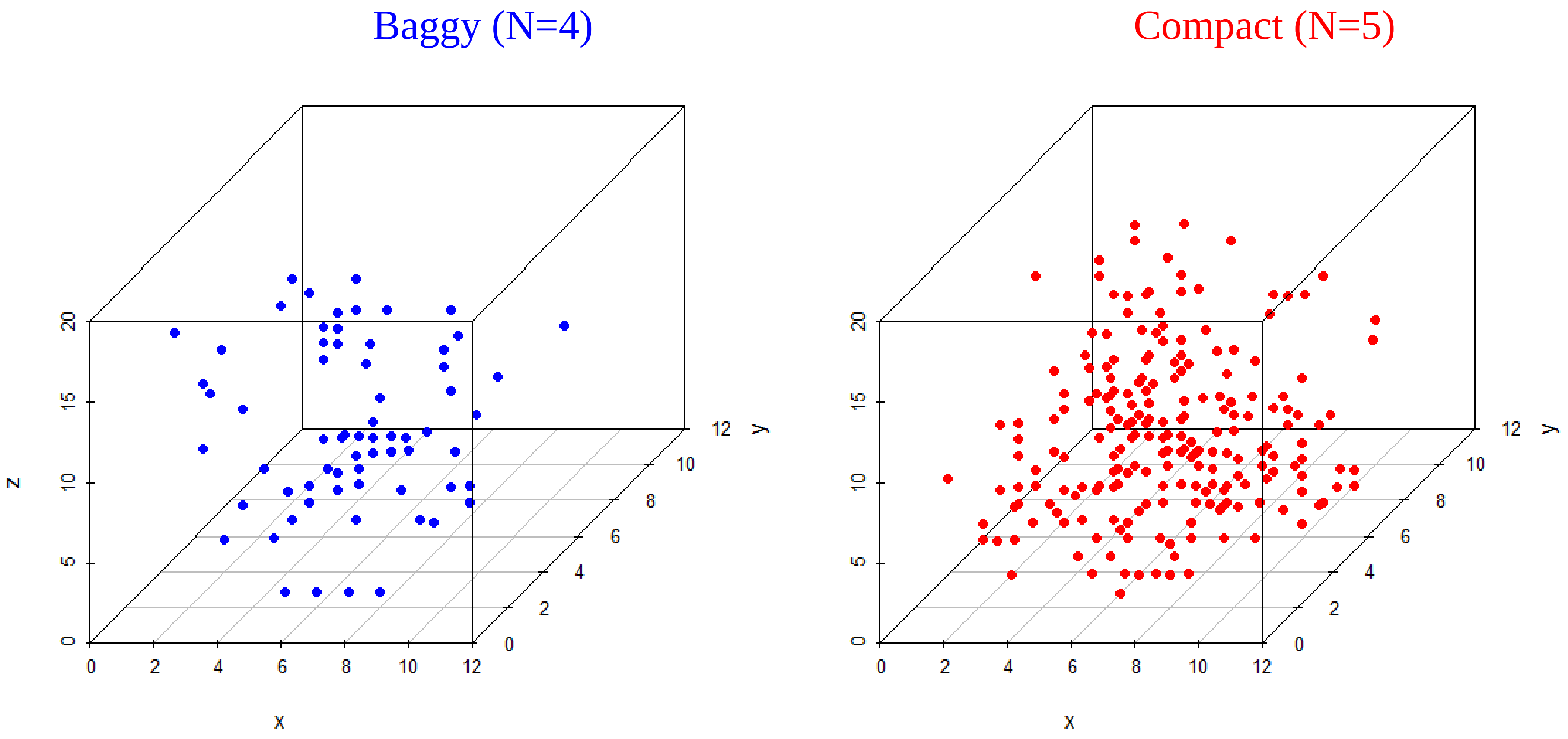

Stretch-bend (1-3 pulls) (8th hour)

Baggy (N=4)

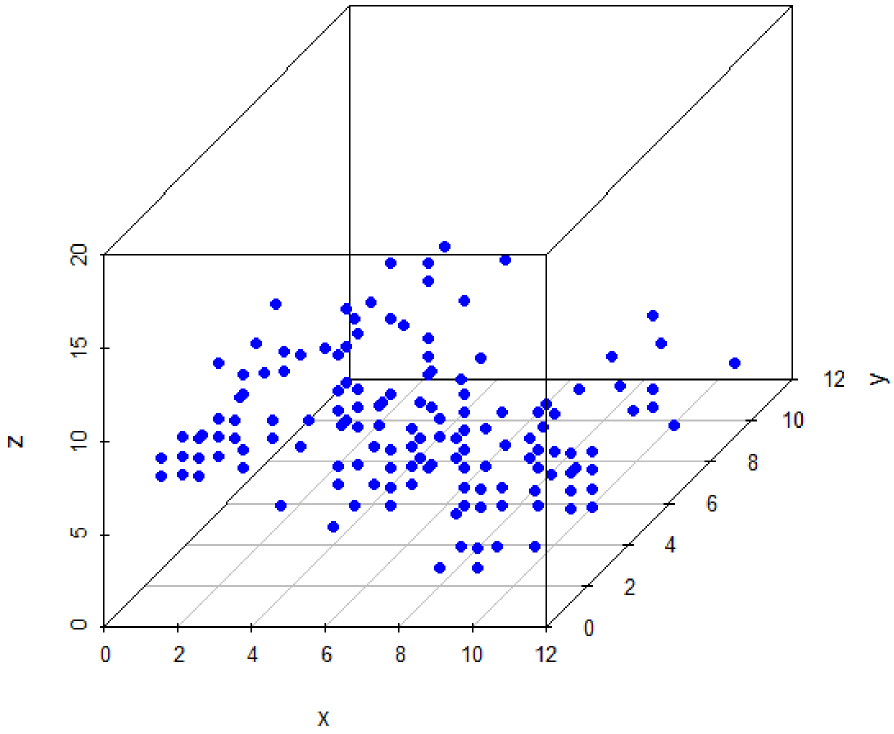

Compact (N=5)

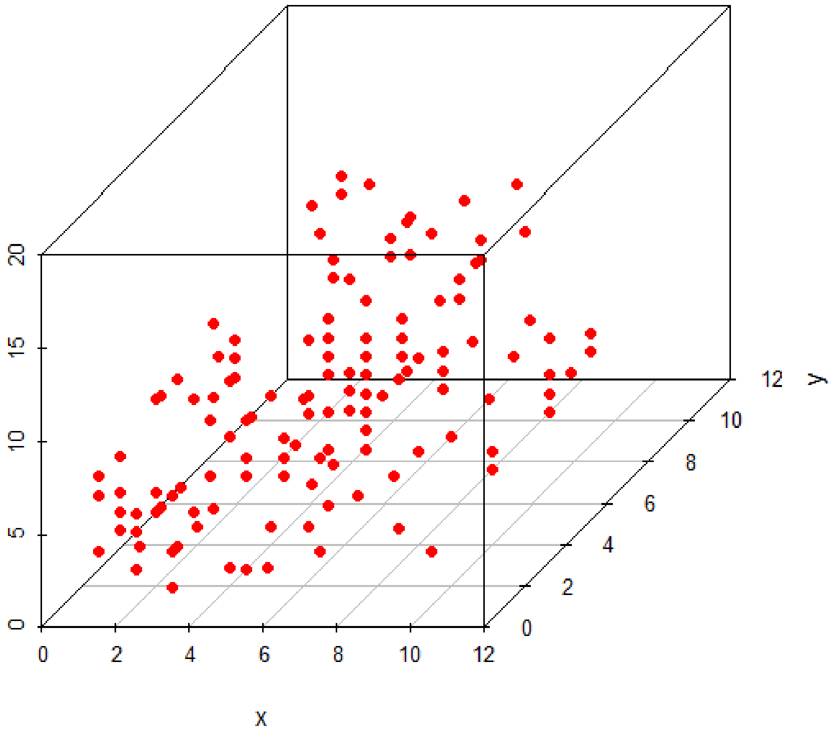

Stretch-bend (1-3 pulls) (12th hour)

Baggy (N=4)

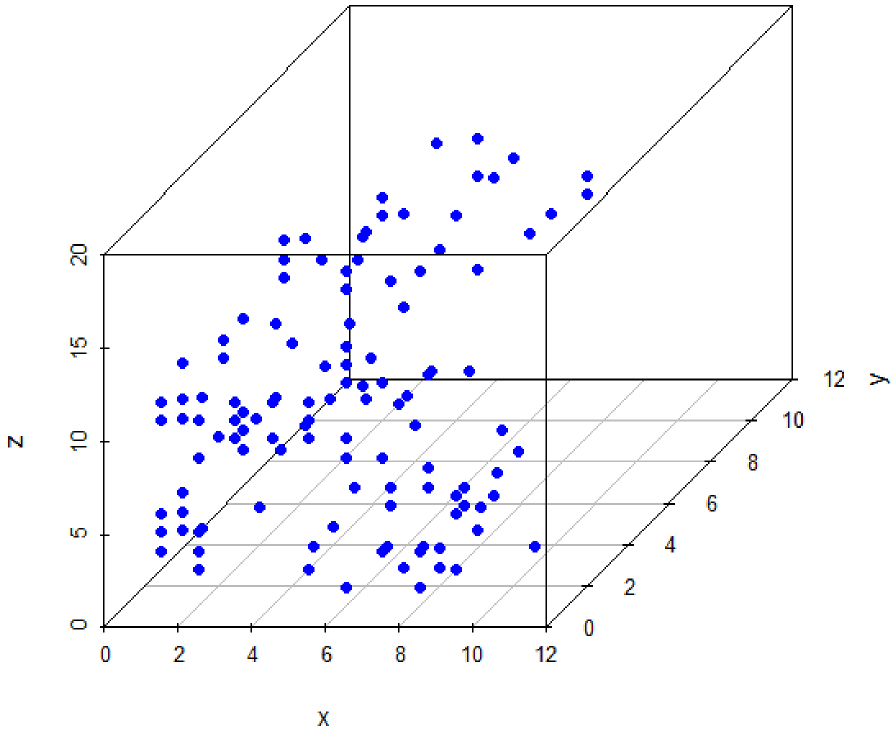

Compact (N=5)

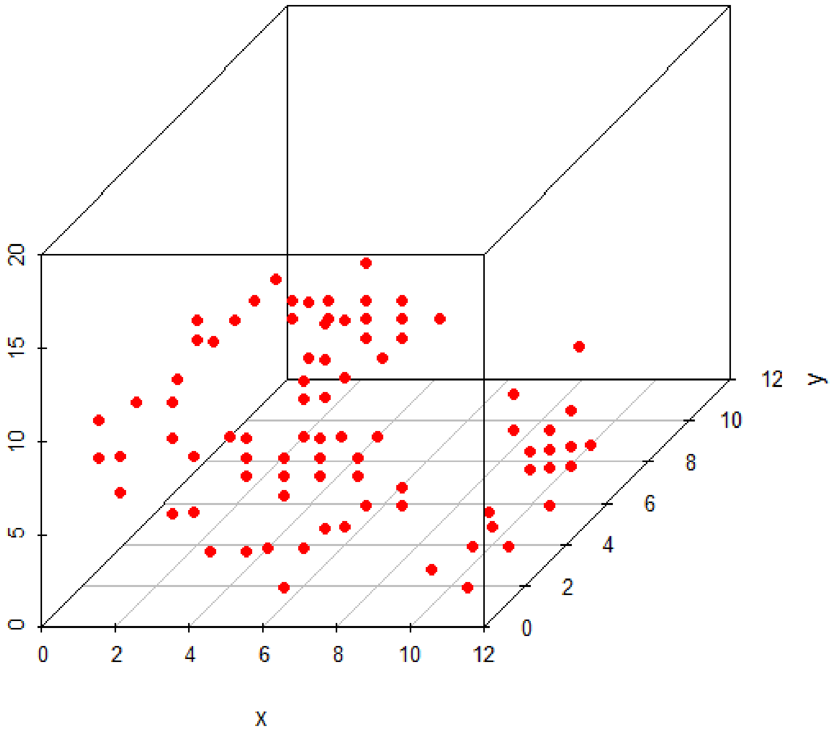

Stretch-bend (1-3 pulls) (16th hour)

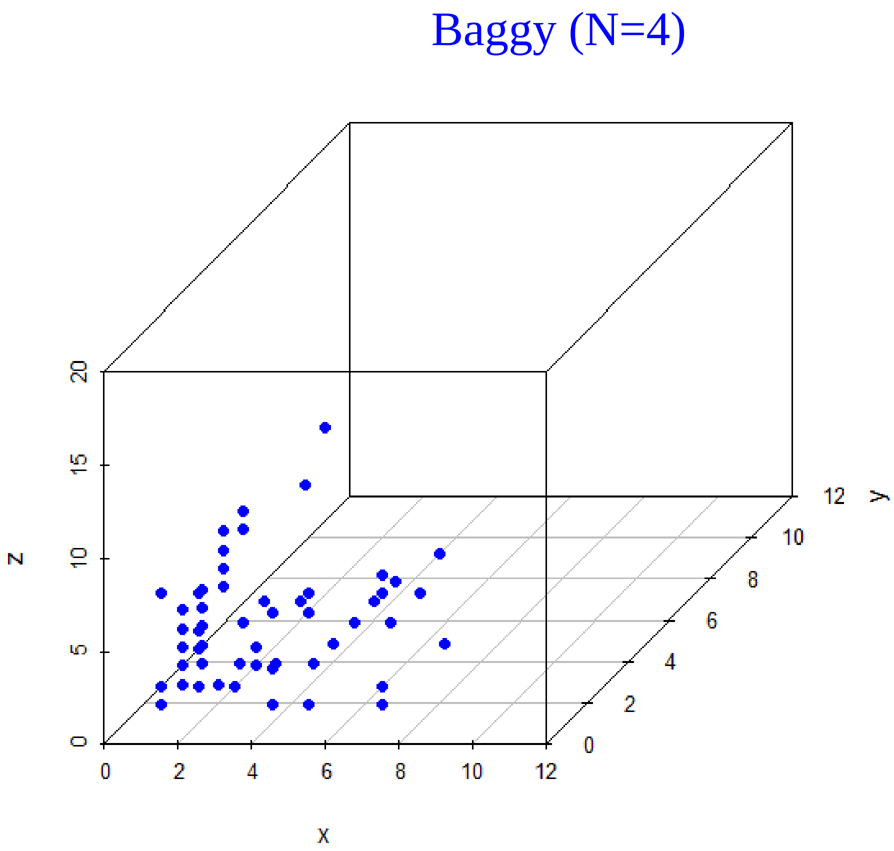

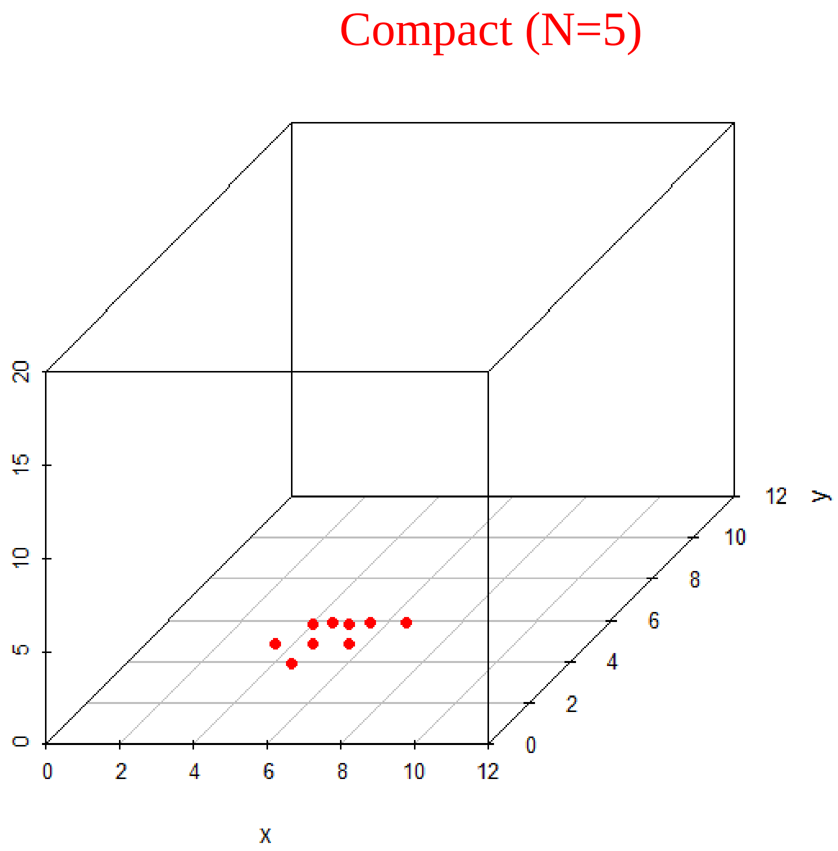

Stretch-bend (>3 pulls) (1st hour)

Baggy (N=4)

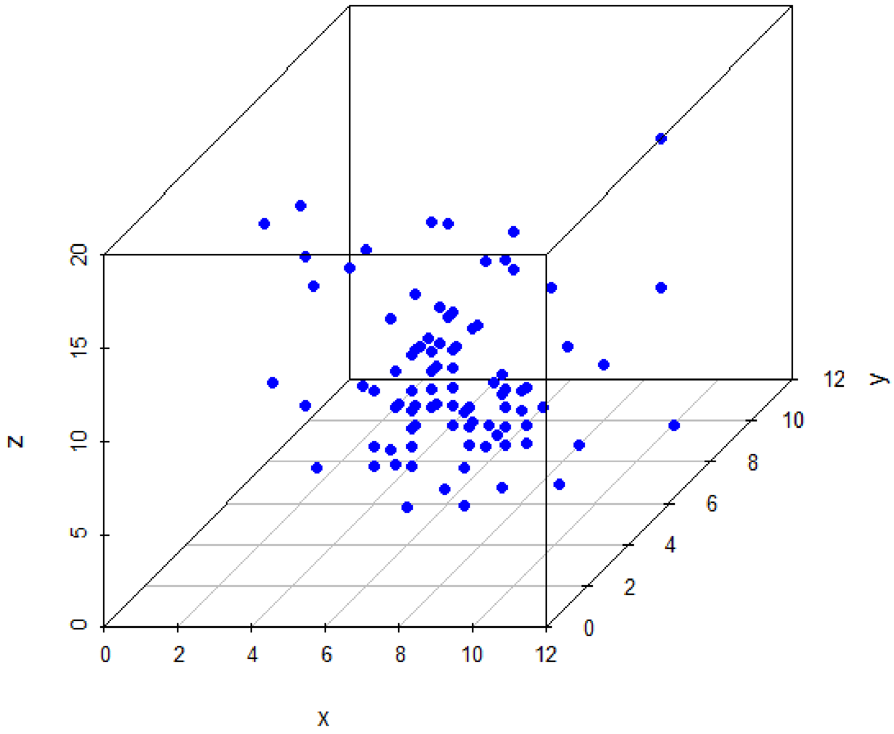

Compact (N=5)

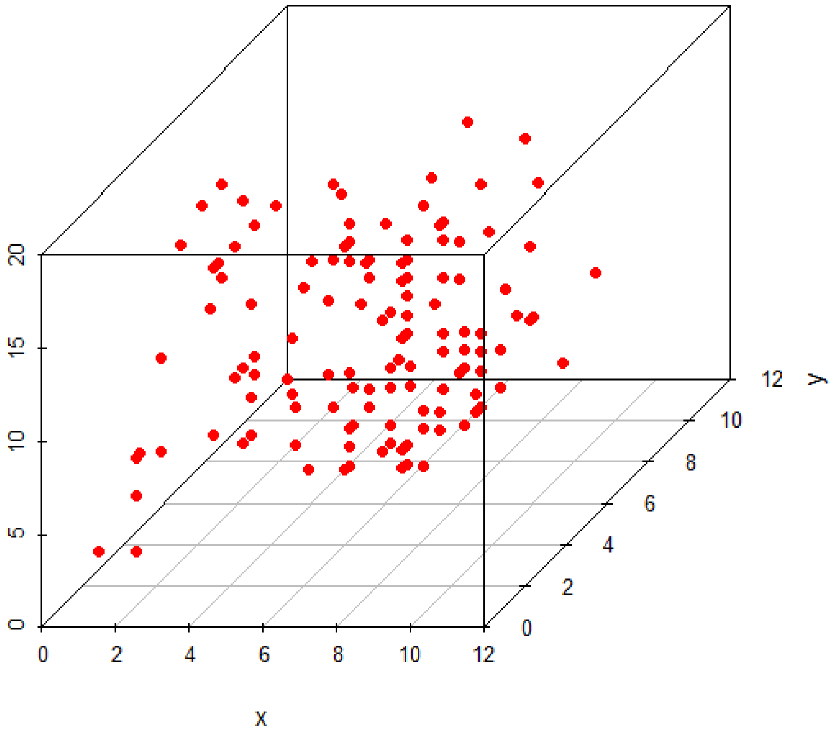

Stretch-bend (>3 pulls) (4th hour)

Baggy (N=4)

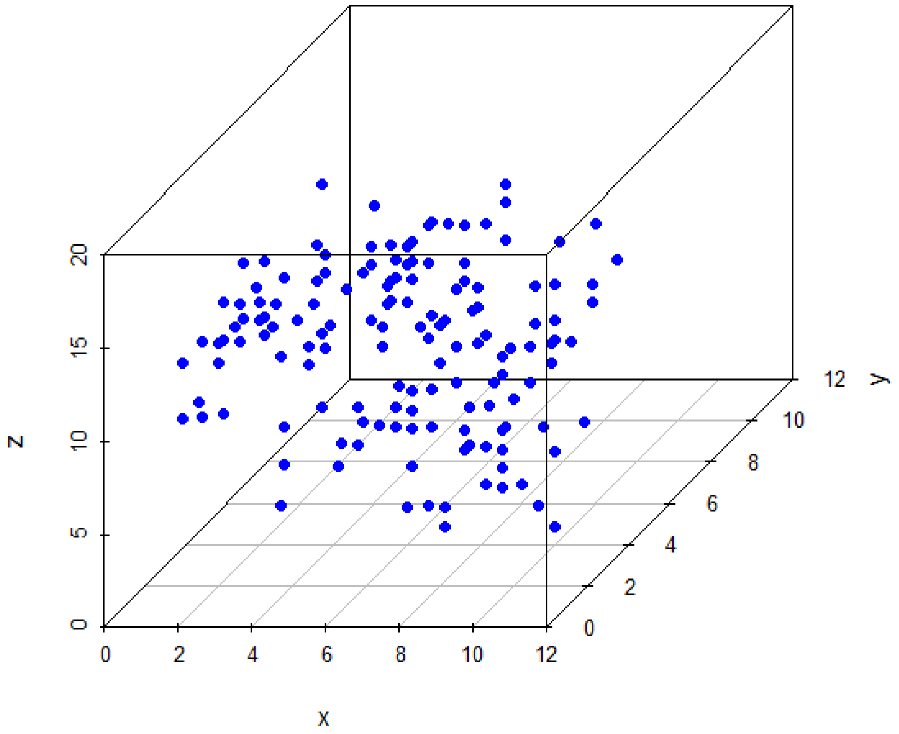

Compact (N=5)

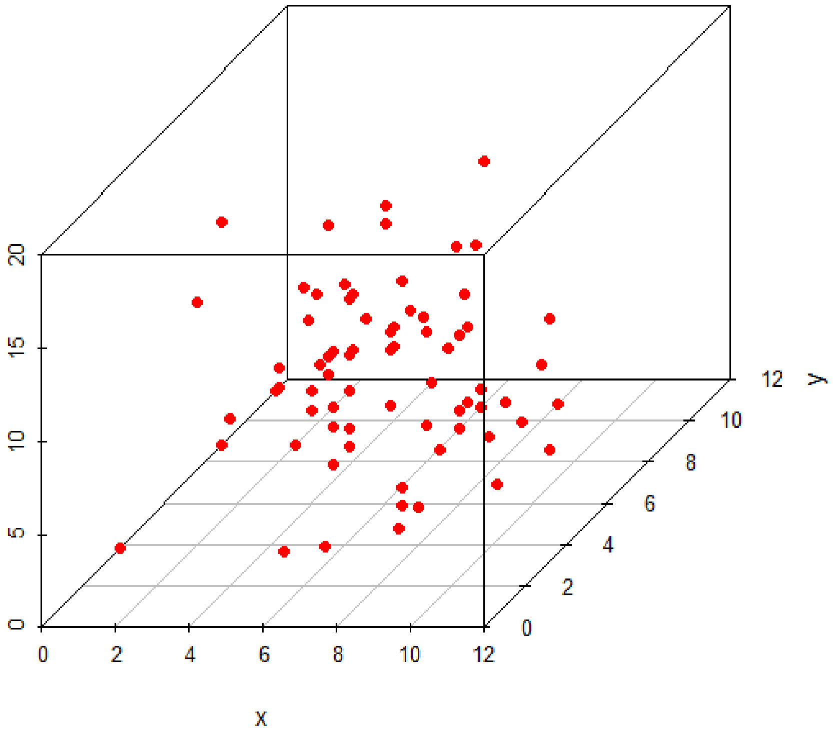

Stretch-bend (>3 pulls) (8th hour)

Baggy (N=4)

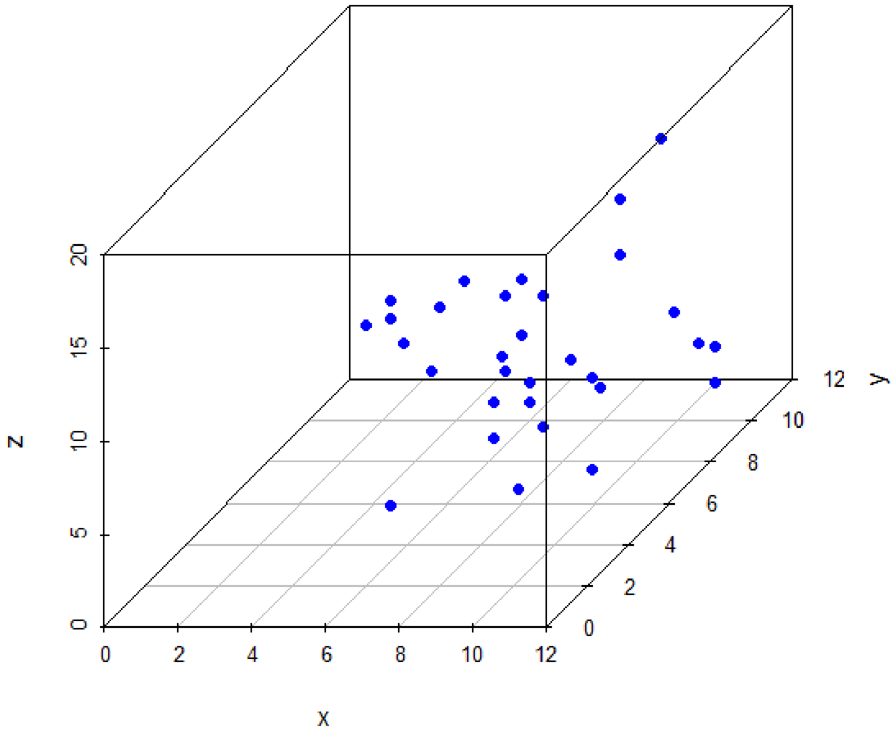

Compact (N=5)

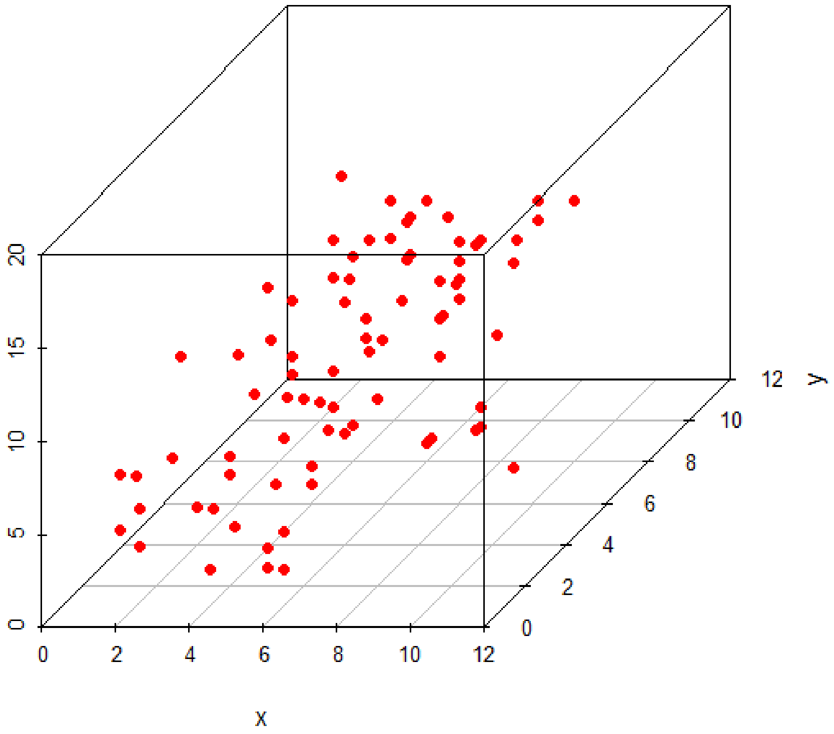

Stretch-bend (>3 pulls) (12th hour)

Baggy (N=4)

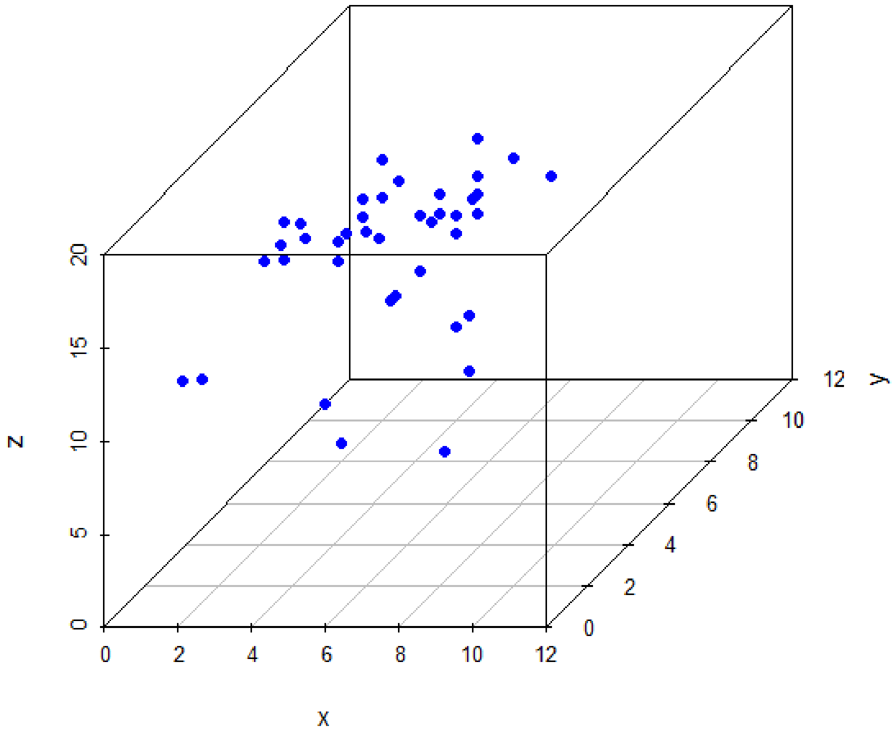

Compact (N=5)

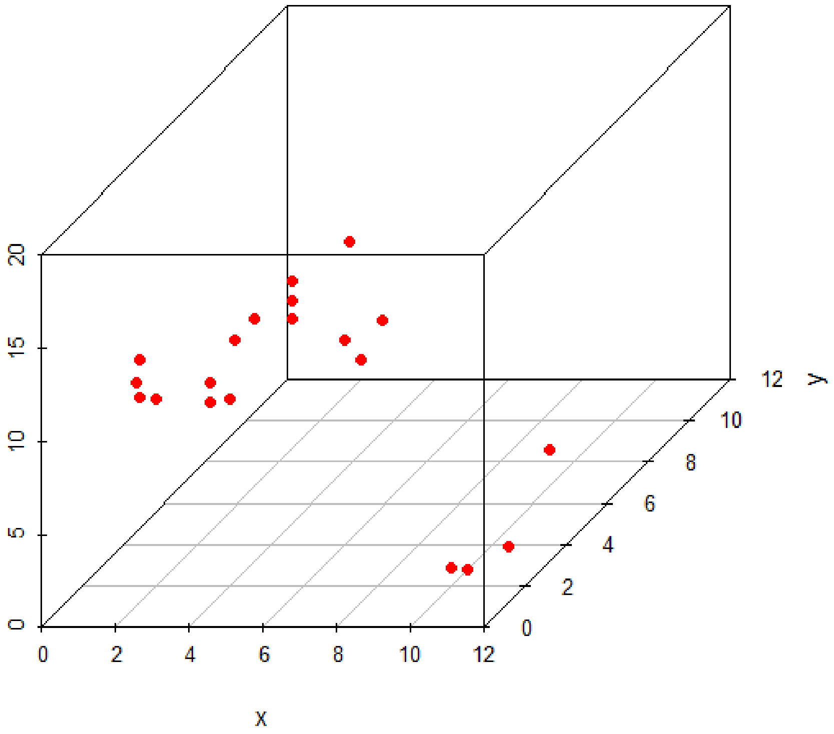

Spinners of both cocoon-types do not perform this behavior during the 16th hour

Swing-swing behavior (1st hour)

Baggy (N=4)

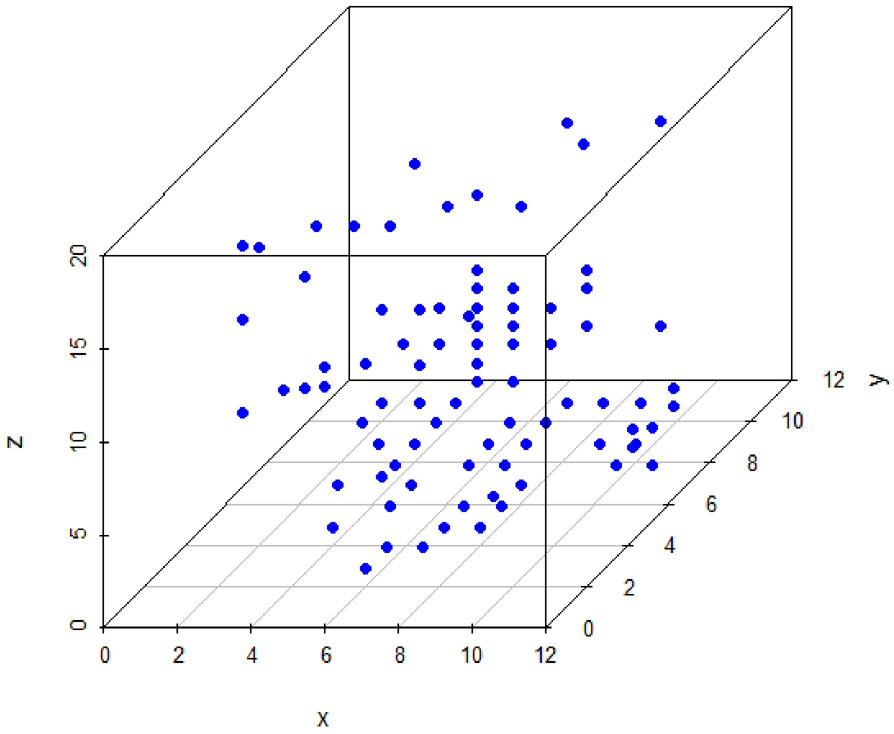

Compact (N=5)

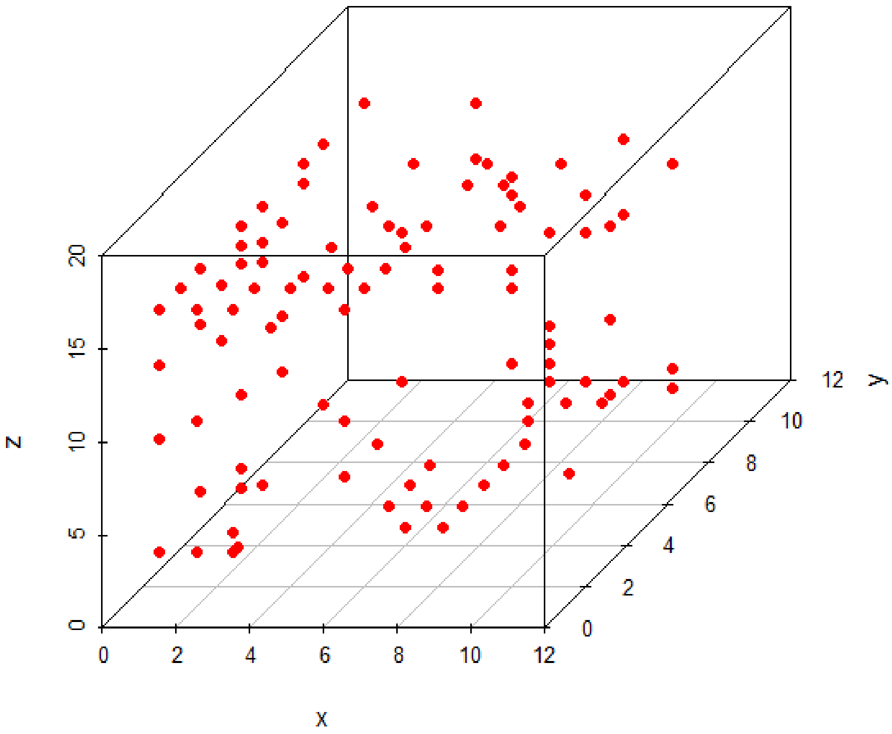

Swing-swing behavior (4th hour)

Baggy (N=4)

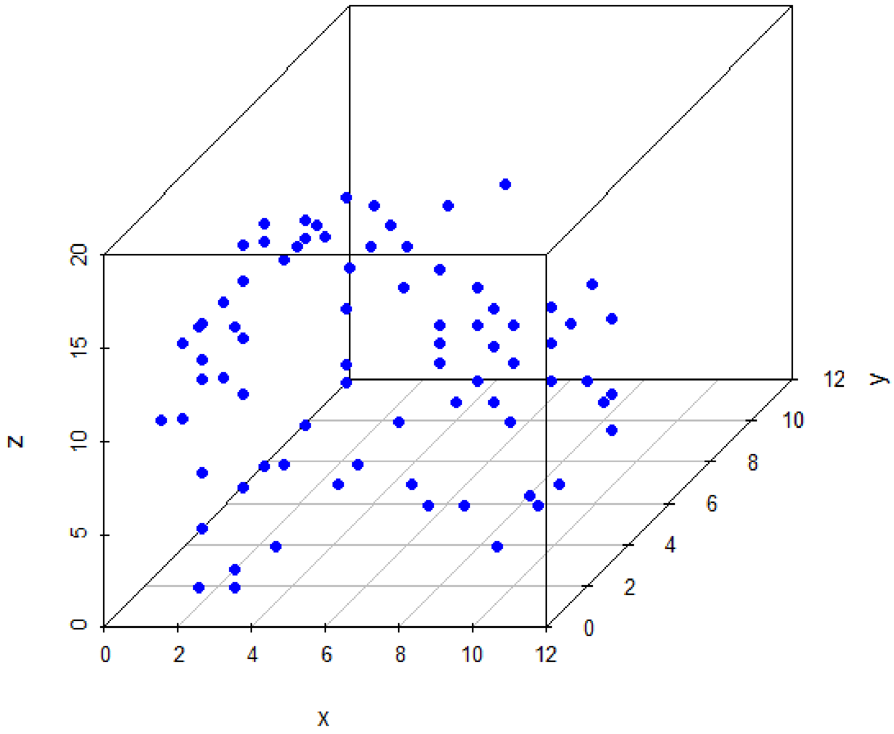

Compact (N=5)

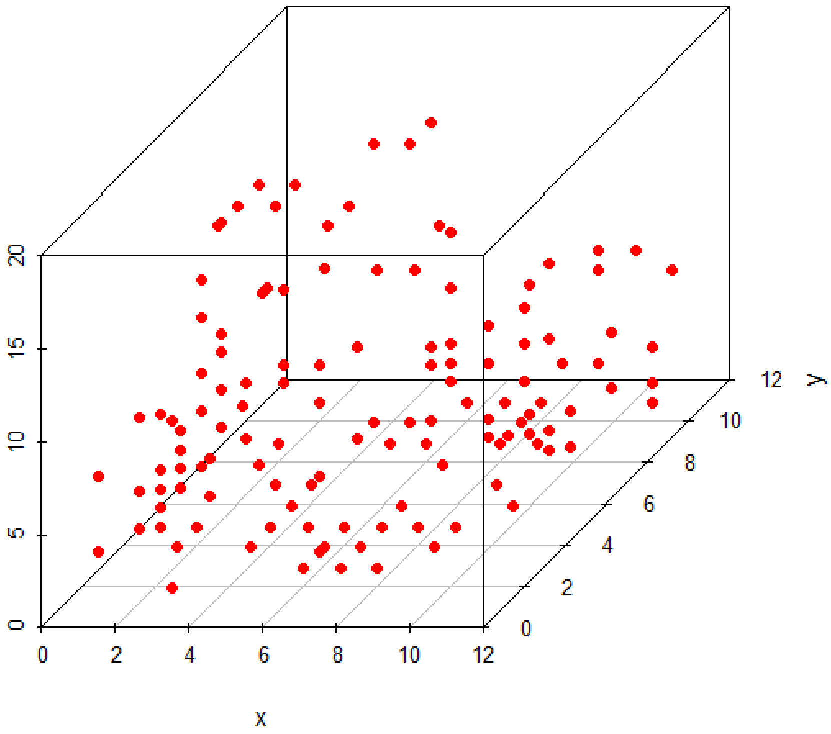

Swing-swing behavior (8th hour)

Baggy (N=4)

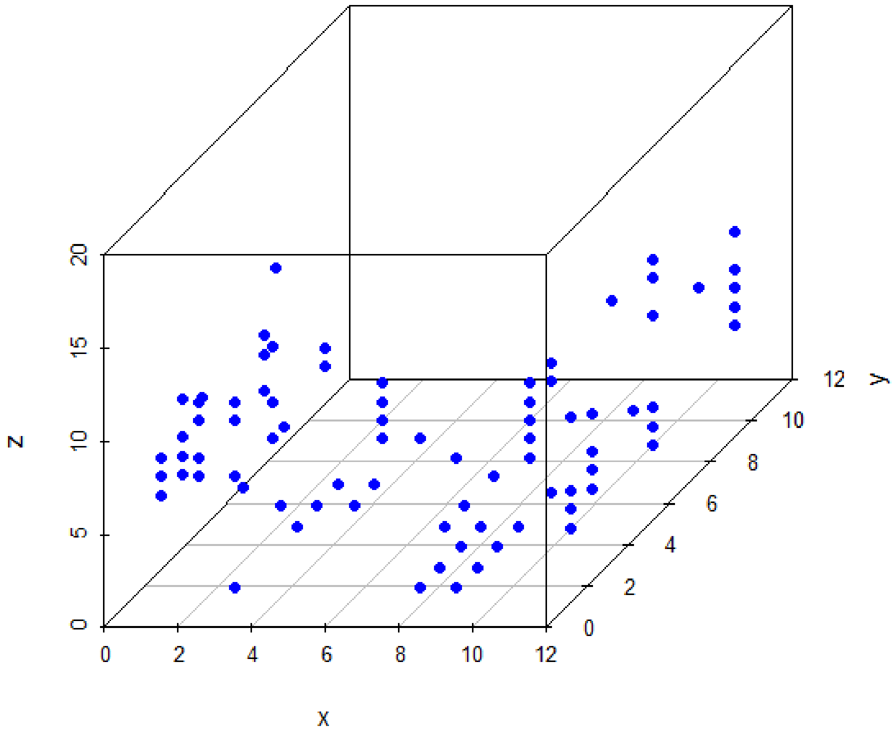

Compact (N=5)

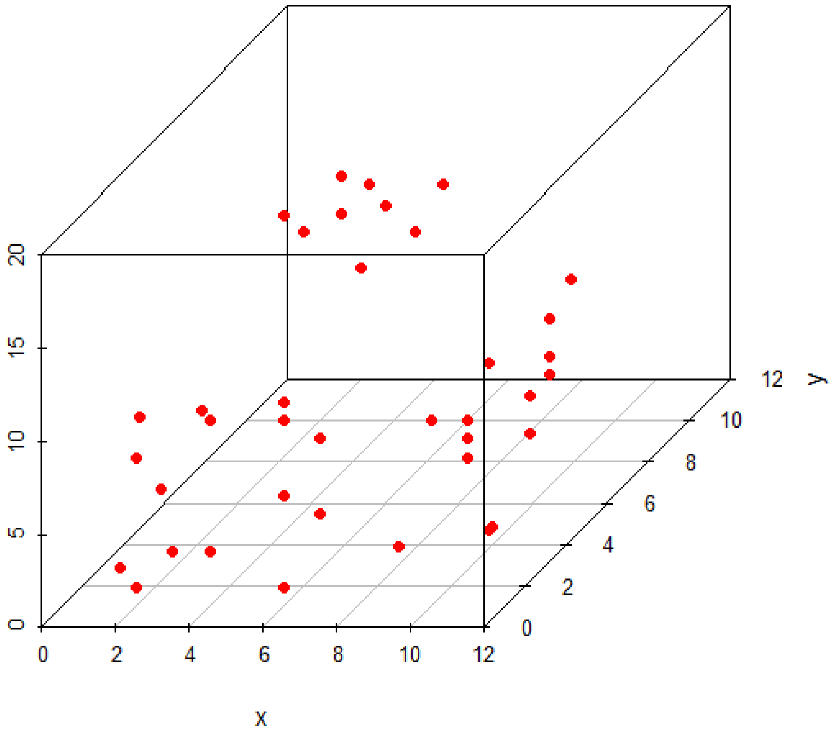

Swing-swing behavior (12th hour)

Baggy (N=4)

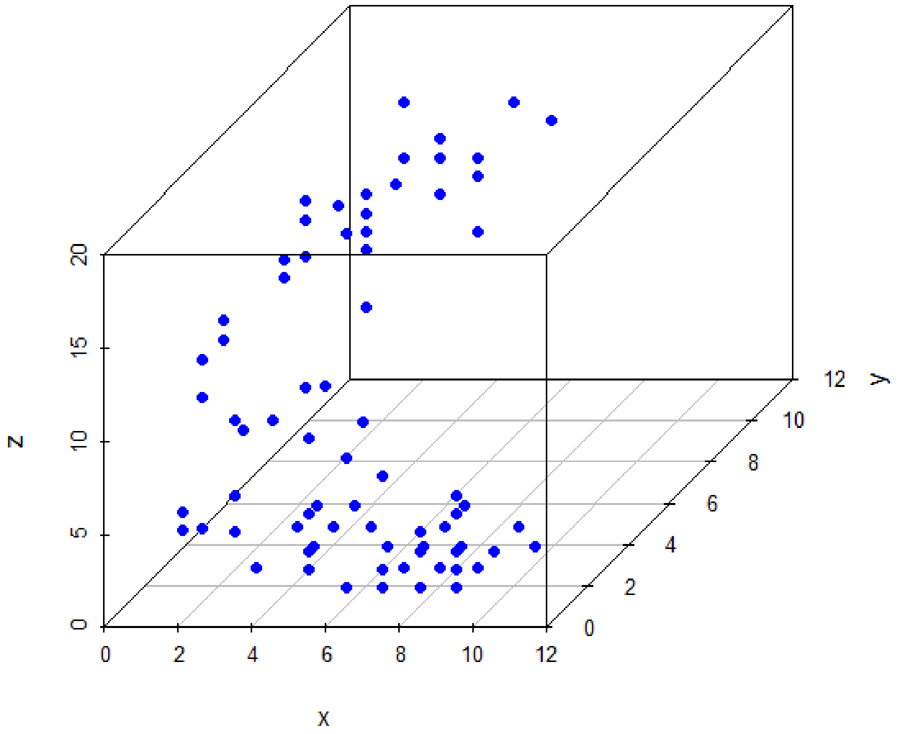

Compact (N=5)

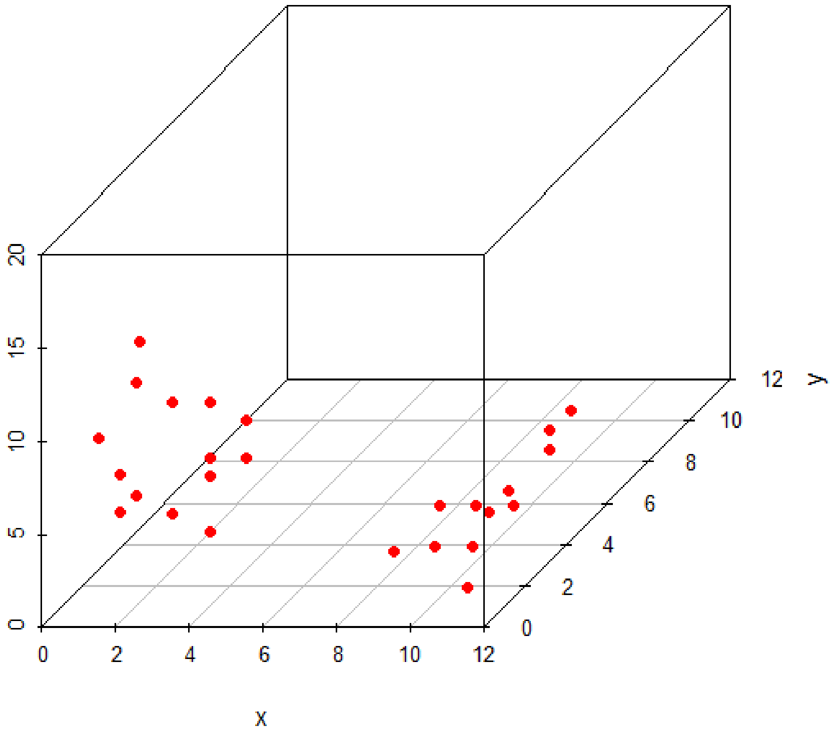

Swing-swing behavior (16th hour)

Baggy (N=4)

Compact (N=5)

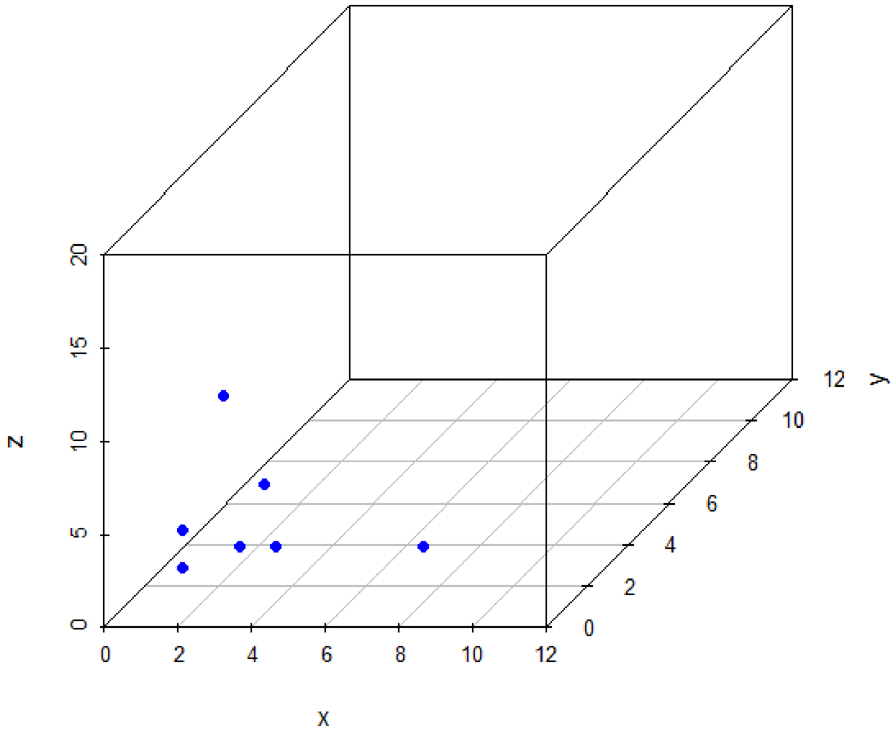

Figure-8 (scaffold stage) behavior (1st hour)

Baggy (N=4)

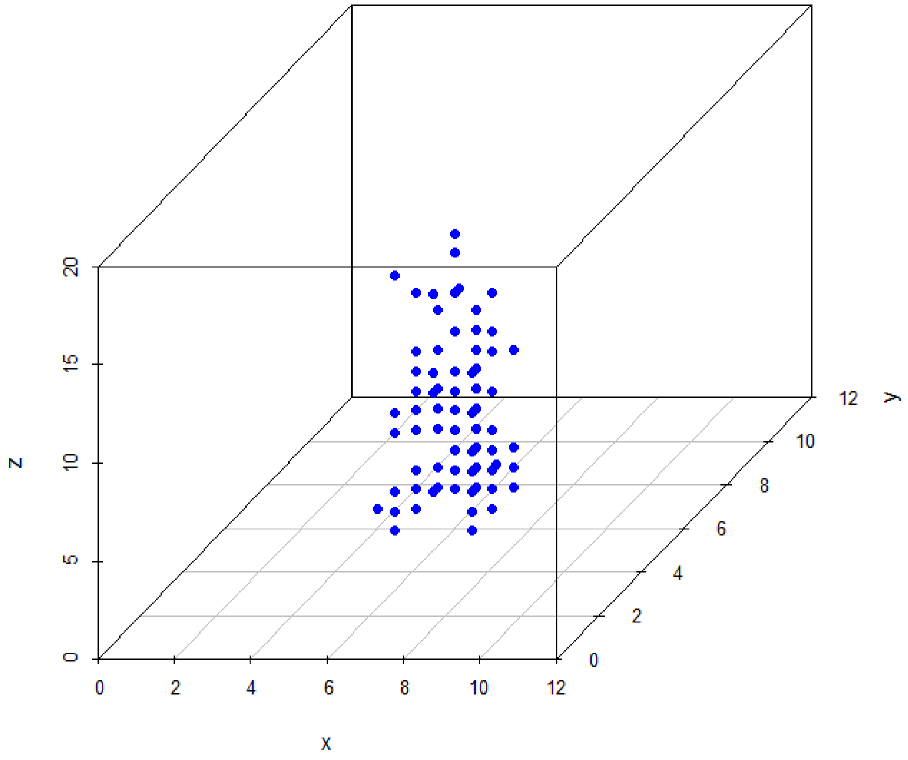

Compact (N=5)

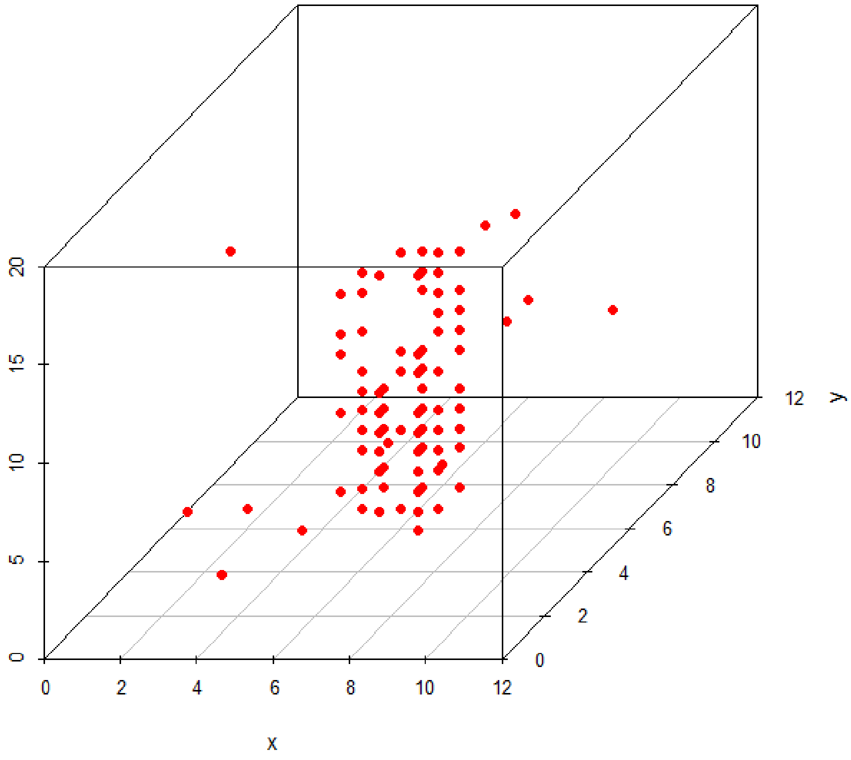

Figure-8 (scaffold stage) behavior (4th hour)

Baggy (N=4)

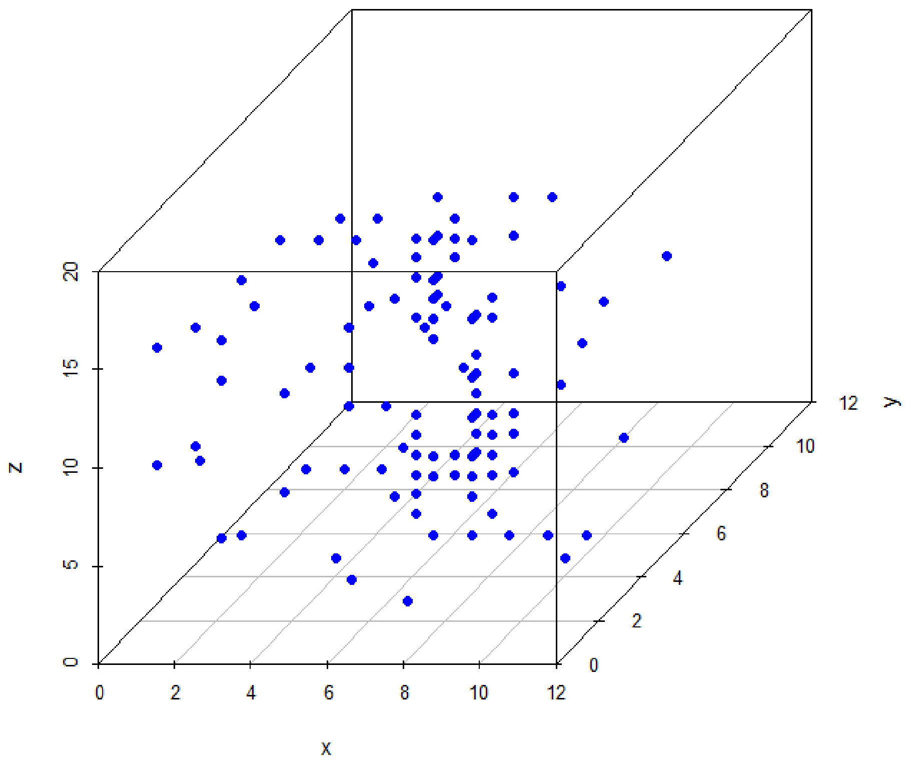

Compact (N=5)

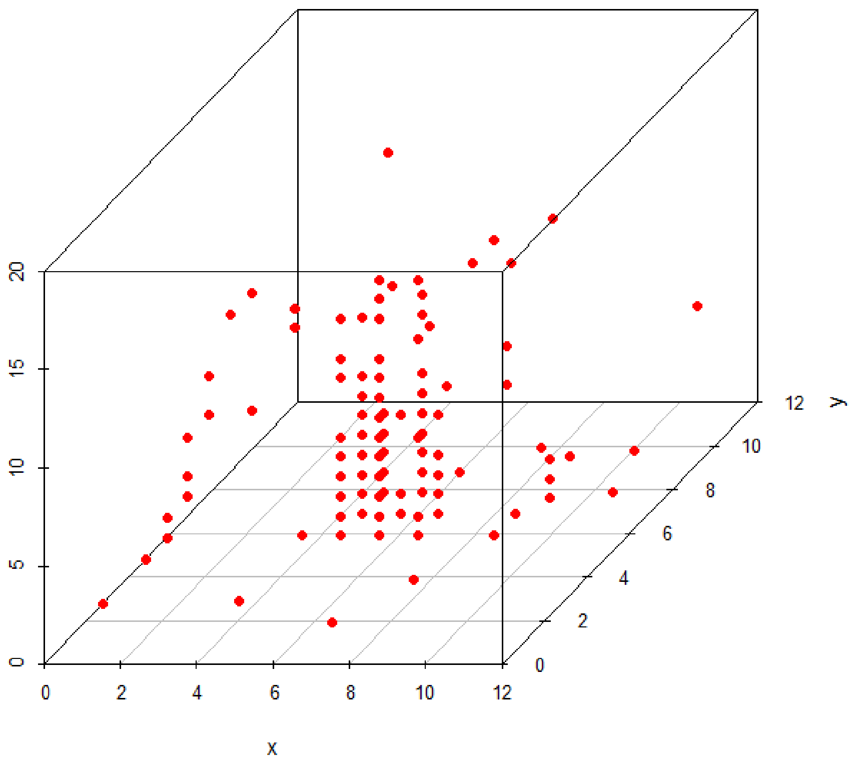

Figure-8 (scaffold stage) behavior (8th hour)

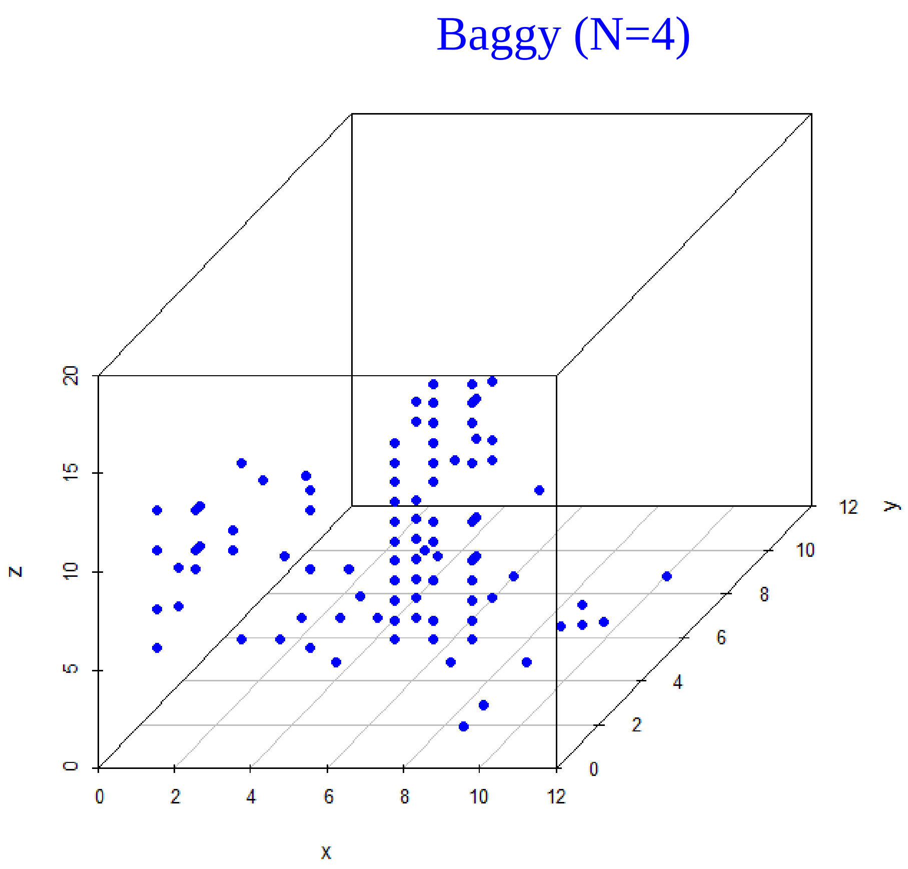

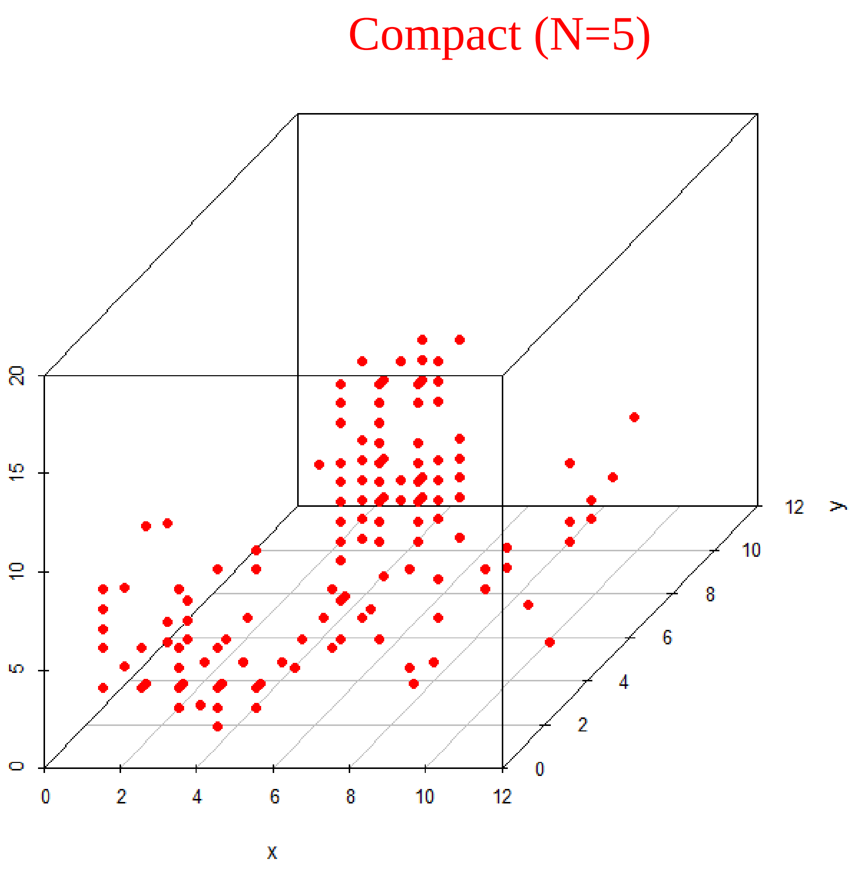

Figure-8 (scaffold stage) behavior (12th hour)

Baggy (N=4)

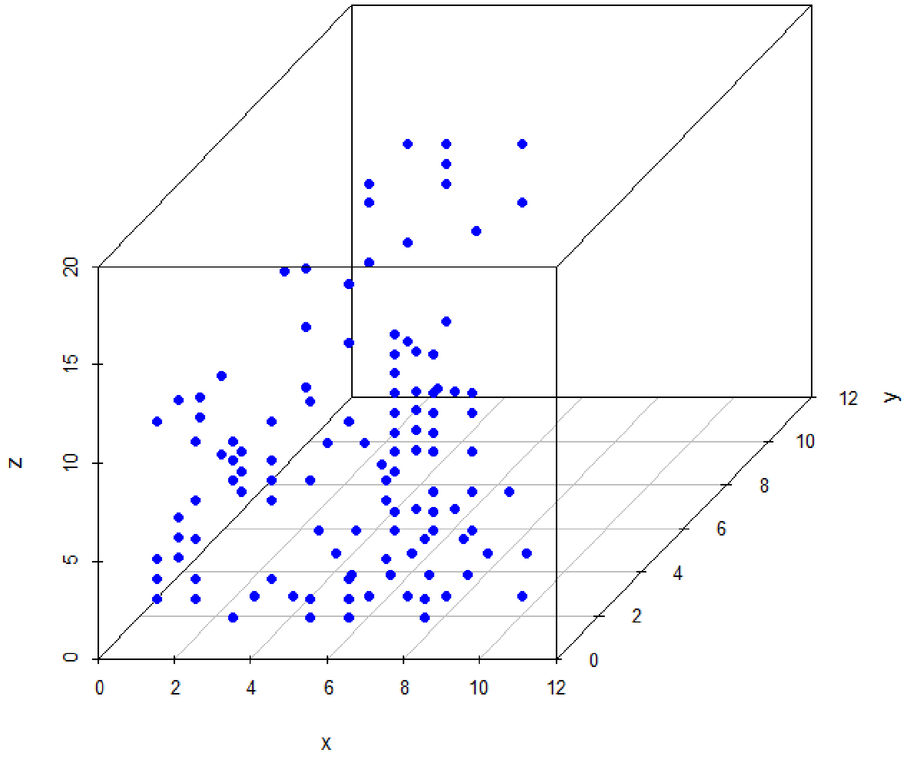

Compact (N=5)

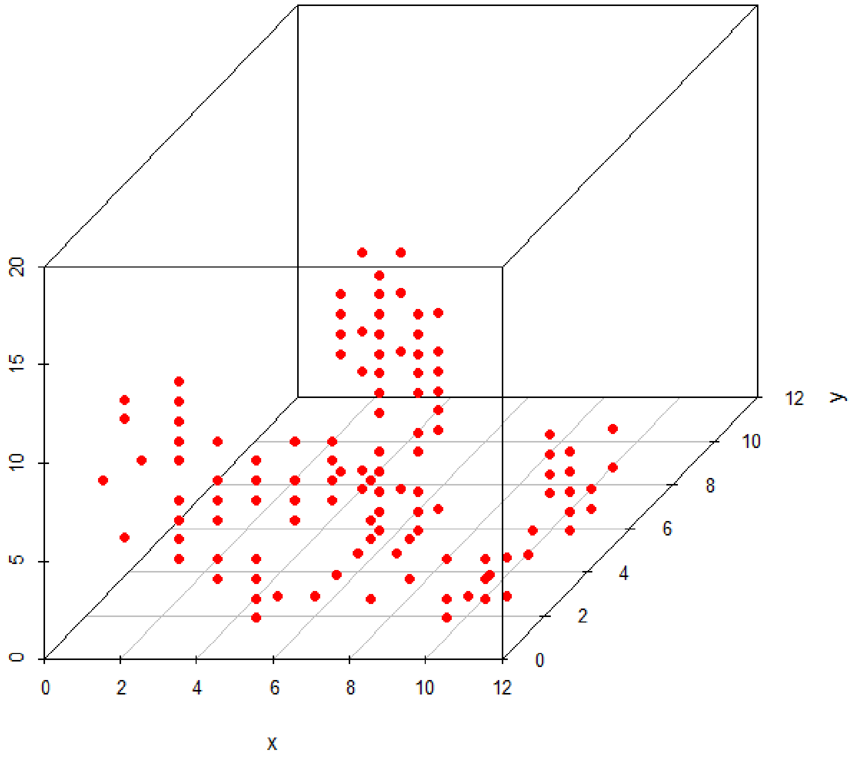

Figure-8 (scaffold stage) behavior (16th hour)

Baggy (N=4)

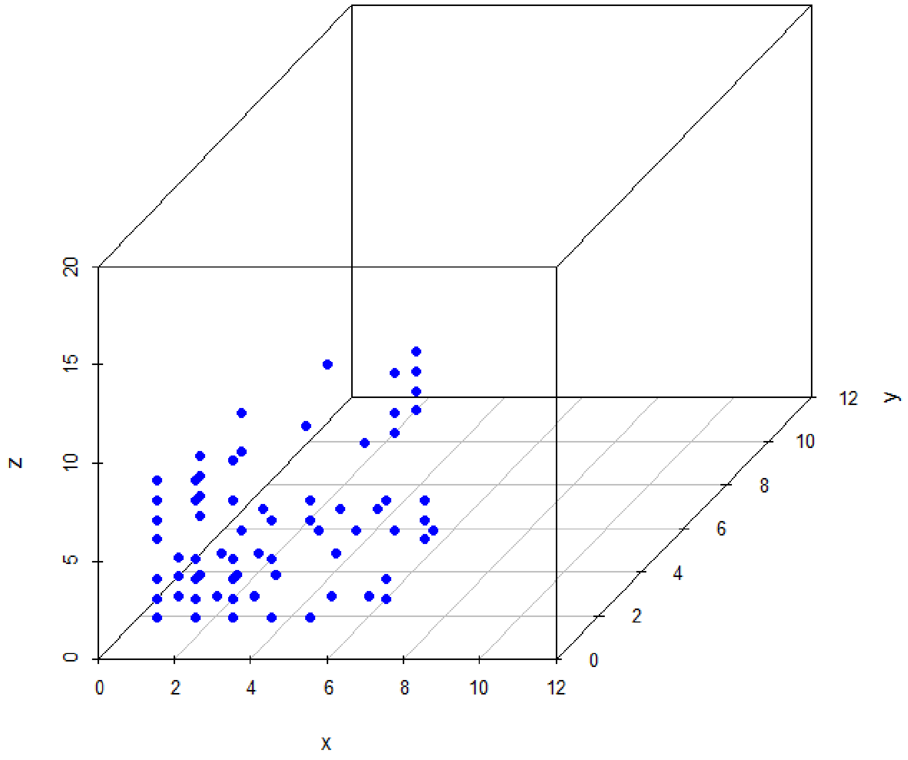

Compact (n=5)

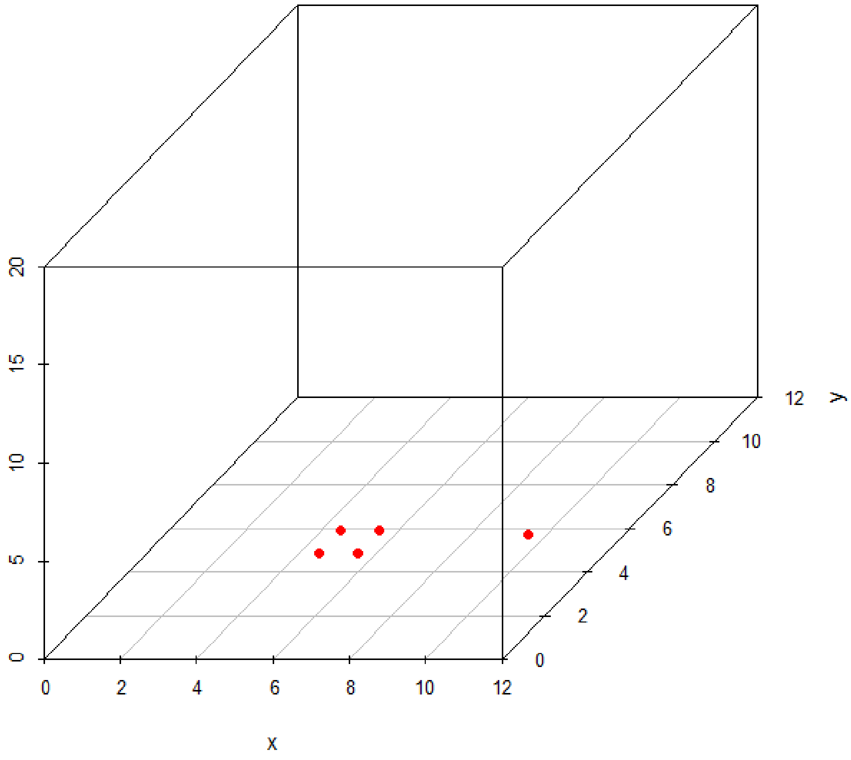

Supplement: S2 Fig — Scatterplots show the location in the arena at which the three major cocoon spinning behaviors (stretch-bend, 1–3 and >3 pulls; swing-swing; figure-8 motion during silk scaffold stage) were performed by spinners of both cocoon-morphs, across all sampling periods. Each dot represents a single behavioral event. Baggy cocoon spinners denoted in blue and compact cocoon spinners denoted in red. (PDF) [file pone.0174023.s009.pdf]
